# A spotlight on multidrug-resistant *Salmonella* in global poultry production

**DOI:** 10.3389/fmicb.2026.1839142

**Published:** 2026-07-01

**Authors:** Dany Mesa, Stella Almeida, Thaís Muniz Vasconcelos, Danieli Conte, Derly Rojas Cruz, Luiza Souza Rodrigues, Adriele Celine Siqueira, Libera Maria Dalla Costa, Lorena Bavia

**Affiliations:** 1 Faculdades Pequeno Príncipe, Av. Iguaçu, 333, CEP 80230-020, Rebouças, Curitiba, PR, Brazil; 2 Instituto de Pesquisa Pelé Pequeno Príncipe, Av. Silva Jardim, 1632, CEP 80250-060, Água Verde, Curitiba, PR, Brazil; 3 Facultad de Medicina Veterinaria y Zootecnia, Universidad Nacional de Colombia, Bogotá, Colombia; 4 Laboratório Central do Estado do Paraná (LACEN-PR), R. Sebastiana Santana Fraga, 1395, CEP 83060-500, Guatupê, São José dos Pinhais, PR, Brazil

**Keywords:** beta-lactamase, multidrug resistance, pESI plasmid, poultry, *Salmonella* Infantis

## Abstract

Salmonellosis is among the most common foodborne infections worldwide, posing a significant economic and health burden. Poultry meat is a primary risk factor for human salmonellosis. The mass production and distribution of poultry meat have contributed to the spread of pathogenic *Salmonella*. Combined with antimicrobial resistance and the rise of multidrug-resistant (MDR) *Salmonella* infections due to animal housing conditions and the excessive and incorrect use of antimicrobial drugs in humans and animals, these factors create new challenges for food safety control. *Salmonella* shows resistance to several antimicrobial classes, including aminoglycosides, beta-lactams, macrolides, tetracyclines, fluoroquinolones, sulfonamides, trimethoprim, fosfomycin, and phenicol antimicrobial classes. This resistance is largely due to *Salmonella*’s ability to acquire and transfer resistance genes, showing high potential for dissemination and representing a significant threat to the effective treatment of salmonellosis. Therefore, this narrative review aims to compile and analyze information regarding the distribution of the main MDR *Salmonella* serovar isolates (excluding Pullorum and Gallinarum) of worldwide poultry production and resistant antibiotic classes, focusing on poultry meat and related products.

## 1 Introduction

The genus *Salmonella* is a global cause of diseases, ranging from relatively mild gastroenteritis to potentially life-threatening invasive infections. This complex genus of bacteria comprises only two species: *Salmonella enterica* and *Salmonella bongori*, with *S. enterica* being the most clinically relevant (Jajere, 2019). *S. enterica* is divided into six subspecies: (I) enterica, (II) salamae, (IIIa) arizonae, (IIIb) diarizonae, (IV) houtenae, and (VI) indica. The genus *Salmonella* includes approximately 2600 different serovars, with approximately 1500 belonging to *S. enterica* subsp. enterica (Chattaway et al., 2021; Lamas et al., 2018). Currently, the name of the serovar is given in non-italicized Roman alphabet letters with the first letter capitalized. Therefore, the full name would be, for example, *Salmonella enterica* subsp. enterica serovar Typhi. Subsequent mentions of the name can be condensed with “*Salmonella*” being followed by just the serovar name, for example, *Salmonella* Typhi or *S.* Typhi (Guibourdenche et al., 2010).

Although most serovars exhibit little host specificity, certain *Salmonella enterica* subsp. enterica serovars are preferentially associated with specific hosts, causing diseases. For example, *Salmonella enterica* subsp. enterica serovar Dublin (*S.* Dublin), is commonly found in cattle (Velasquez-Munoz et al., 2024), while *S.* serovars, Pullorum and Gallinarum, are typically found in chickens (Farhat et al., 2024). Other serovars, such as *S.* Typhimurium, cause enteric diseases in several hosts, including songbirds (Hernandez et al., 2012). Furthermore, *S.* Typhimurium has been implicated in human infections (Perez-Sepulveda and Hinton, 2025).

*Salmonella*, a foodborne pathogen, contaminates various food-producing animals, such as poultry, turkey, sheep, pigs, fish, and cattle (Barrow and Methner, 2013). Specifically, poultry and poultry products are considered an important source of *Salmonella* infection in humans (Wigley, 2024). Nonetheless, *Salmonella* can contaminate soil, water, crops, fruits, vegetables, and surfaces (Jechalke et al., 2019; Liu et al., 2018).

Although easily transmitted by animals and environmental factors, non-typhoidal *Salmonella* (NTS) infections have a low incidence in adults and in countries with higher incomes (Crump et al., 2015). However, *Salmonella* infections have a higher prevalence in middle- and low-income countries (Nazir et al., 2025). According to the global burden of invasive disease, NTS caused 535,000 cases, leading to 77,500 deaths, while typhoid and paratyphoid fever accounted for around 14.3 million cases and approximately 136,000 deaths (Stanaway et al., 2019). The average global annual consumption of antibiotics in animal production is estimated at 172 mg/kg and 148 mg/kg for pigs and poultry, respectively, with rising global consumption (Van Boeckel et al., 2015; Tiseo et al., 2020).

Although antimicrobials may be used for non-therapeutic purposes, the use in non-therapeutic conditions can lead environmental bacteria to adapt and acquire resistance genes (Larsson and Flach, 2022), commonly transferable between different bacterial genera, spreading several resistance genes to enteropathogens, such as *Salmonella* (Pokharel et al., 2006), in the community context (Antunes et al., 2016; Klemm et al., 2018). The spread of antimicrobial resistance genes (ARGs) from other bacteria to *Salmonella* has led to the emergence of MDR strains in poultry (Davalos et al., 2025; Lee et al., 2025; Huang et al., 2025; Saggin et al., 2025). Importantly, the definition most frequently used for MDR Gram-negative bacteria is resistant to three or more antimicrobial classes (Magiorakos et al., 2012).

This narrative review aims to assess the distribution of MDR *Salmonella* serovars and resistant antibiotic classes across continents to better understand the dynamics of antimicrobial resistance in *Salmonella* in the world poultry industry, focusing on poultry meat and related products, primarily researching since the 2000s.

## 2 Search strategy

This narrative review obtained data from a search conducted in English across the PubMed, Scopus, Google Scholar and Web of Science databases to identify peer-reviewed studies investigating MDR *Salmonella* in poultry. Searches were performed using variations of the following search function: (“multidrug-resistant *Salmonella*” OR MDR *Salmonella*) AND (poultry OR chicken). The inclusion criteria encompassed studies published in the last 26 years (between February 2000 and February 2026) that related MDR strains of *Salmonella* isolated from poultry. The determined period is a limitation of this narrative review, considering that a deep and critical meta-analysis is not the focus of this document that rather emphasizes an overview on the subject. Articles regarding *Salmonella* serovars, Pullorum and Gallinarum were excluded. Conference abstracts were discarded as well. Articles were selected by two reviewers initially based on the title and abstract.

## 3 Antibiotics used in the poultry industry and related resistance genes

This review considers information on antimicrobial drug resistance used in the poultry industry. The wide range of available antimicrobials are categorized by classes as follows: aminoglycosides (e.g., streptomycin, gentamicin, amikacin), beta-lactams (e.g., penicillin, amoxicillin, cephalosporins, carbapenems), macrolides (e.g., erythromycin, tylosin), tetracyclines (e.g., oxytetracycline, tetracycline, chlortetracycline), fluoroquinolones (e.g., ciprofloxacin, enrofloxacin, nalidixic acid), sulfonamides (e.g., sulfamethoxazole, sulfadiazine), folate synthesis inhibitor (e.g., trimethoprim), fosfomycin (e.g., fosfomycin), nitrofurans (e.g., furazolidone, nitrofurazone, nitrofurantoin), and phenicols (e.g., chloramphenicol, thiamphenicol, florfenicol). ARGs identified in *Salmonella* strains from the poultry industry across countries are listed in Supplementary Table 1.

Extended-spectrum beta-lactamases (ESBL), mainly CTX-M-type and AmpC-producing in *Salmonella* isolates from humans and animals worldwide, (Huang et al., 2025; Jeon et al., 2019) are rapidly rising. Although AmpC genes are intrinsically found in *Salmonella*, they appear due to acquired mechanisms. The AmpC beta-lactamase *bla*_*CMY–*2_, commonly found on plasmids, encodes resistance to cefoxitin (second-generation cephalosporin antibiotic) and is a significant source of β-lactam resistance in *Salmonella* (Cejas et al., 2014). The *bla*_*CMY–*2_-containing plasmids can harbor resistance genes to other classes of antimicrobials, potentially conferring MDR (Edirmanasinghe et al., 2017). Another common antimicrobial resistance profile observed in *Salmonella* isolates is resistance to ampicillin, streptomycin, sulfonamides, tetracyclines, and fluoroquinolones, which has increased and may be associated with poultry sources (Kipper et al., 2022; Mesa et al., 2026).

Although the review does not focus on the prevalence of ARGs or the distribution of plasmids, some of the selected articles provide these data (Bogomazova et al., 2020; Campos et al., 2018; Martínez-Puchol et al., 2021). In general, genes encoding resistance to sulfonamide, aminoglycosides, quinolones, and β-lactams are the most prevalent in Brazil, Canada, China, and England (Bai et al., 2016; Bharat et al., 2022; Biffi et al., 2014; Davies et al., 2022). However, in some cases, similar antimicrobial-resistant bacteria have been observed, regardless of the serovar. For example, bacteria in Portugal and Italy exhibited the same resistance profile, with different dominant serovars (Campos et al., 2018). In turn, studies from Cambodia and Bangladesh showed the same resistance profile, identifying resistance to polymyxin (Parvin et al., 2022).

The World Health Organization (WHO) has created a list of bacterial pathogens of public health importance to guide research, development, and strategies for preventing and controlling antimicrobial resistance. Inclusion of *Salmonella* and *Shigella* under high priority reflects their increasing resistance to existing treatments and the high burden of infection associated with these pathogens, particularly in low- and middle-income countries (World Health Organization [WHO], 2024). The ongoing prevalence of antimicrobial resistance has led the CDC to classify the threat level of drug-resistant non-typhoidal *Salmonella* as “serious” (CDC, 2019). In addition, the international trade of animal protein has significantly contributed to the spread of ARGs, often serving as a potential reservoir for *Salmonella* MDR (Jung et al., 2021). Some studies have suggested an association between the consumption of imported chicken meat and the incidence of salmonellosis (Davies et al., 2022; Abdi et al., 2017; Al-Hadidi et al., 2022; Nadimpalli et al., 2018). However, others report conflicting resistance patterns (Campos et al., 2018; Davies et al., 2022; Abdi et al., 2017).

**FIGURE 1:**
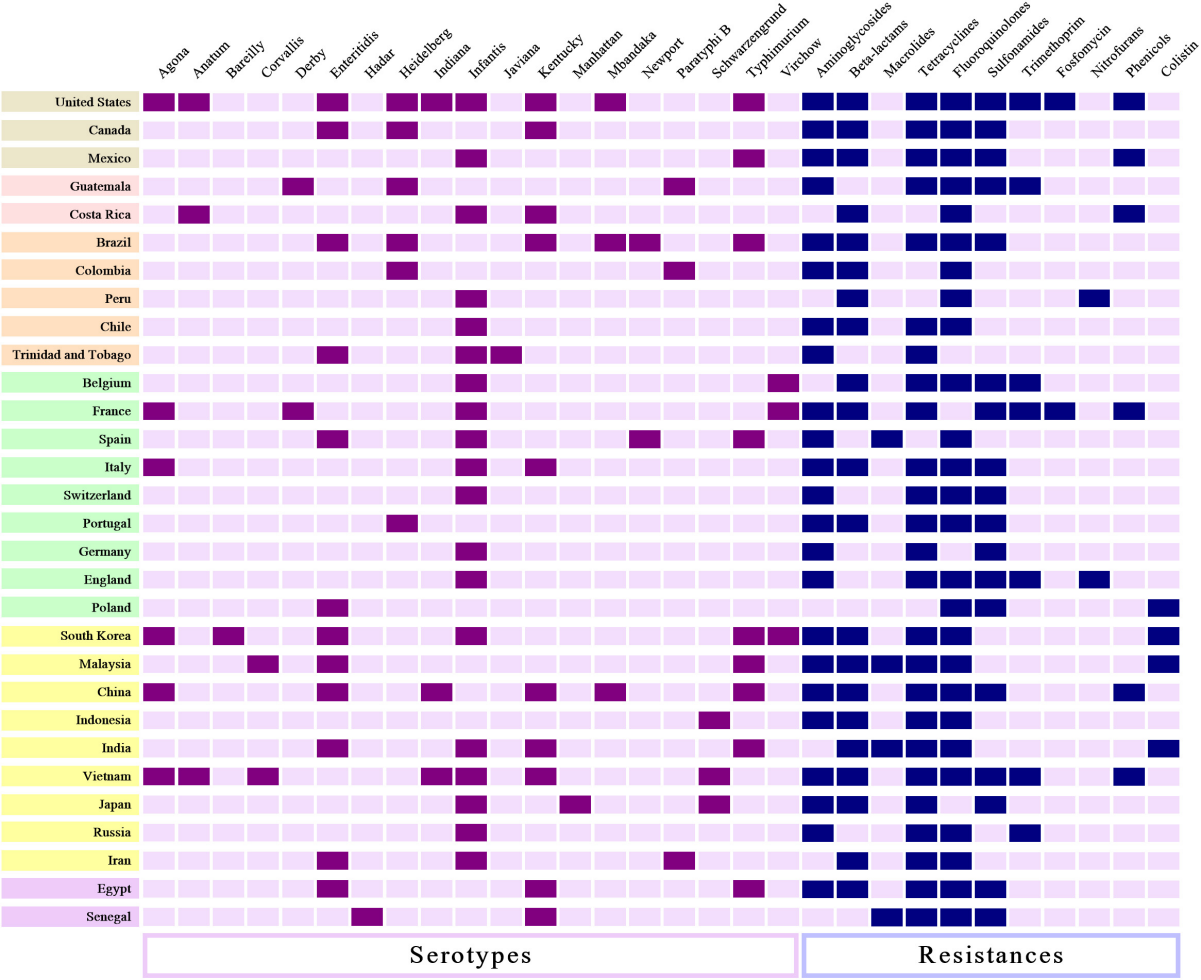
Distribution of main MDR *Salmonella* serovars and classes of antimicrobials among different countries worldwide. Countries of the same color mean they are from the same continent. This figure was created using GraphPad Prism 8.

**FIGURE 2:**
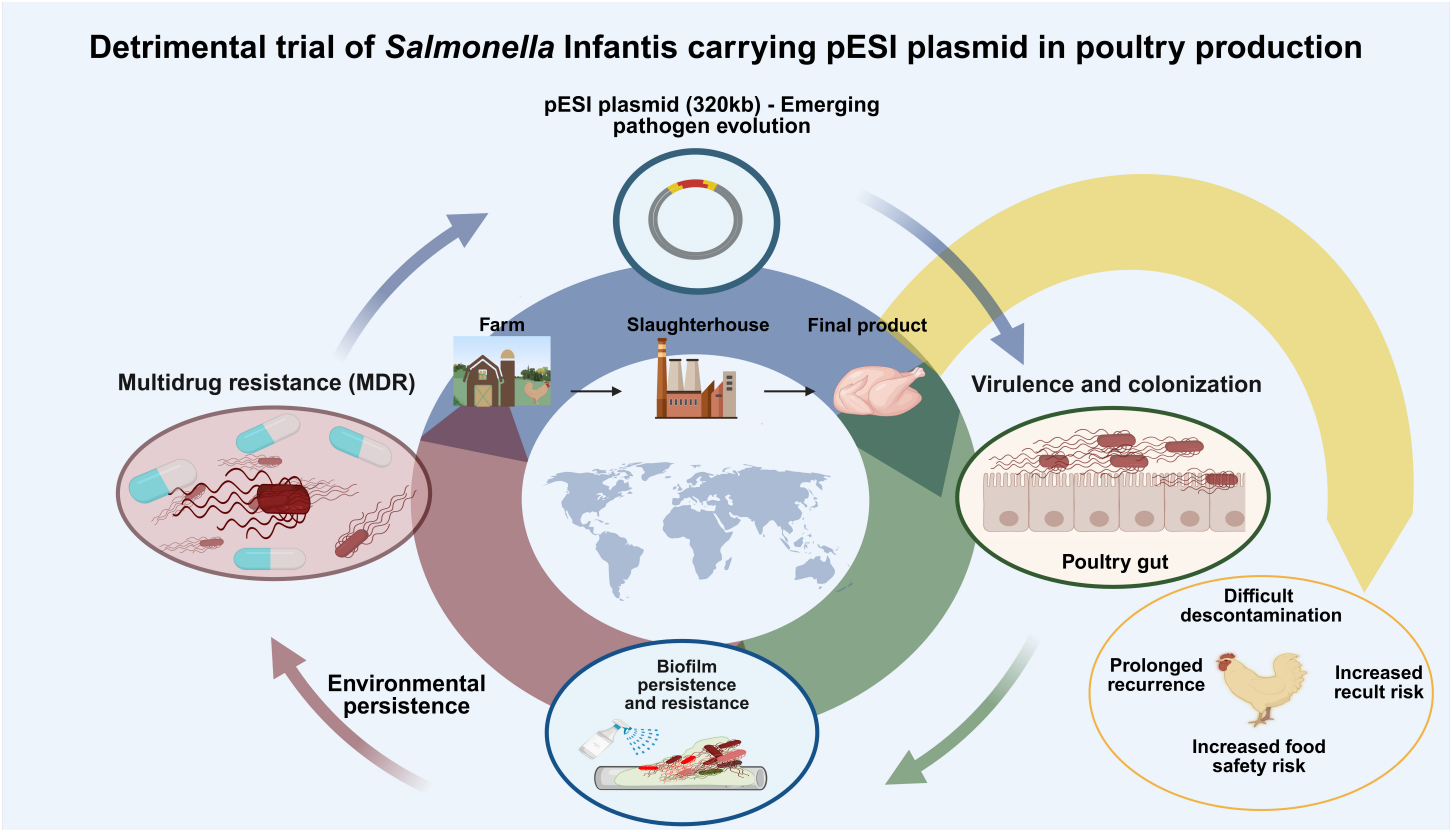
Detrimental trial of *S.* Infantis carrying pESI plasmid in poultry production. This figure was created using Biorender.com.

Considering the extensive trade in poultry products and the issue of the spread of MDR *Salmonella*, we organized the information by continent to present a global overview. A summary of the main MDR *Salmonella* serovars and antimicrobial classes by country is provided in Figure 1. Additionally, in Figure 2, detrimental triad mechanism of *S.* Infantis carrying pSEI plasmid in poultry production is shown.

### 3.1 America

#### 3.1.1 North America

Canada is among the largest countries in the world, with a long-running investigation over poultry-related *Salmonella* infections, since NTS infection is related to 87,510 food-related illnesses, among over 900 hospitalizations and 17 deaths per year (Kanoatova et al., 2024). While Salmonellosis and *Salmonella* were first associated with raw consumption or even uncooked frozen poultry-derived products, data from 2000 to 2010 already indicated *Salmonella* spp. as one of the leading causes of foodborne domestically acquired illness. Data from 2007 to 2021 suggest that *Salmonella* isolates from poultry layers in the years 2009–11 were frequently resistant to tetracyclines 5.1% (8/117 isolates), and along the years, every isolate expressed resistance to at least one class of antimicrobial (Medrano et al., 2024).

A more recent paper from Caffrey et al. (2021) showed that from 2013 to 2018, isolates from both chicken and turkey broilers (*n* = 1596) were more likely to be resistant to clinically important antimicrobials, such as cephalosporins (174/1596) and fluoroquinolones (41/1596), also pointing to high levels of tetracycline resistance, present in over half of the isolates. MDR was found in 231 out of the 1596 isolates, with streptomycin, tetracycline and beta-lactam resistance, the most frequent pattern. Three serovars, *S.* Kentucky (*n* = 573, 36%), *S.* Enteritidis (*n* = 314, 20%) and *S.* Heidelberg (*n* = 127, 8%) were the most abundant (Caffrey et al., 2021). Corroborating this data, ESBL-producing *Salmonella* were already reported in 2022 in Bharat’s genomic analysis, after screening over 30,000 surveillance isolates collected by the Canadian Integrated Program for Antimicrobial Resistance Surveillance (CIPARS); authors found 22 isolates from turkey (meat or animal) and 3 isolates from chicken, harboring ESBL class A resistance, of which resistance to ampicillin, ceftiofur, ceftriaxone and gentamicin were the most common (28%/7 out of 25 isolates). MDR isolates were found most predominantly in *S.* Albany (7/25) but overall accounted for 13 out of the 25 isolates from poultry, expressing mostly resistance to β lactam, aminoglycoside, sulfonamide or tetracycline (Bharat et al., 2022).

Like Canada, Mexico is one of the largest countries in the world, but with a far less developed surveillance program, leading to scarce and often diffuse, mixed data from samples collected at the end of the production chain and from in-site broiler sampling. As such, Miranda et al. (2009) showed that poultry meat was the most frequent source of *Salmonella* in the year 2009, accounting for 35.3%, or 41 isolates of the 441 samples collected from both supermarkets and wet markets, with the latter reported as more likely to have contaminated meat, a trend that would change over the next decade as shown by Regalado-Pineda et al. (2020) in their longitudinal study, by 2016–18 supermarkets would have contaminated meat with *Salmonella*, with the prevalence of this pathogen rising from 13.7% in 2016 to 27.1% in 2018.

When it comes to antimicrobial resistant *Salmonella* in poultry, most of the studies we found had consistent results; Miranda et al. (2009) found a high prevalence of MDR isolates in Hidalgo State (85.3%), and although the most dominant pattern was ampicillin, chloramphenicol, and tetracycline (11/41), patterns harboring quinolone resistance accounted for a much larger proportion (20/41). This resistance to quinolones in *Salmonella* would once more be confirmed by Godínez-Oviedo et al. (2020) in their study from the years 2000 to 2017, with ampicillin, sulfonamides, tetracycline, and quinolones being the most frequent MDR profile in Mexico from both human and food samples.

Although these phenotypes expressed low frequency of cephalosporin resistance, genomic analysis of *S.* Infantis isolated from 2008 to 2024 throughout 27 Mexican states showed that 121/191 isolates were carrying pESI-like megaplasmids, 117 of those isolates sampled from poultry, pointing to an undetected MDR lineage spreading. The CTX-M-gyrA6-8 genotype present in those plasmids accounted for over 70% of all poultry-isolated *S.* Infantis (88/121), characterized by the presence of *bla*_*CTX–M–*65_ and gyrA d87Y mutation, a globally reported resistance/genomic profile suggesting international dissemination (Delgado-Suarez et al., 2026). While previous research identified *S.* Typhimurium as the dominant MDR serotype in both food and clinical samples, poultry-associated *Salmonella* (Godínez-Oviedo et al., 2020).

In Minnesota, United States, 30% (15/50) of *S.* Typhimurium isolates from feed ingredients or poultry sources exhibited the following antimicrobial resistance pattern: ampicillin, chloramphenicol, streptomycin, sulfonamides, and tetracycline. Among these, the MDR *S.* Typhimurium definitive type 104 (DT104) was identified in 4/7 of feed ingredients and in 7/43 of poultry sources (Rajashekara et al., 2000). In a study from Pennsylvania, 378 chicken meat samples were analyzed, of which 22% (84/378) were positive for *Salmonella* spp. The most frequent serovars were *S.* Typhimurium, *S.* Kentucky, and *S.* Enteritidis. Among them, 31% of *Salmonella* spp. were resistant to three or more antimicrobials, and 21% were resistant to ceftiofur (M’Ikanatha et al., 2010). From a turkey facility, 95 samples were collected, and the most frequent *Salmonella* serovars isolated included *S.* Montevideo, *S.* Anatum, and *S.* Agona. Regarding antimicrobial resistance, 36% of isolates were resistant to two or more antimicrobials, such as sulfisoxazole (36%), gentamicin (26%), streptomycin (18%), tetracycline (12%), and trimethoprim-sulfamethoxazole (11%) (Sanad et al., 2016).

Furthermore, another study evaluated genomes and antimicrobial resistance of five isolates of *S.* Infantis containing the resistance gene, *bla*_*CTX–M–*65_. All isolates were resistant to ampicillin, tetracycline, ceftriaxone, ceftiofur, nalidixic acid, aztreonam, and cefotaxime (Tate et al., 2017). In turn, Velasquez et al. (2018) evaluated the antimicrobial resistance of *Salmonella* spp. in four poultry farms. Of all of *Salmonella* spp. isolates, 68% were resistant to at least one antimicrobial class, 26% to two classes, and 5% to three antimicrobial classes, classified as MDR. The main prevalent serovars were *S.* Enteritidis, *S.* Berta, and *S.* Mbandaka. In turn, Hu et al. (2020) evaluated antimicrobial resistance and genes related to 143 *Salmonella* spp. isolates from chicken and egg sources. Antimicrobial resistance was specific to each *Salmonella* serovar, with *S.* Enteritidis isolates resistant to aminoglycosides, *S.* Typhimurium resistant to aminoglycosides, tetracycline, and sulfonamides, and *S.* Heidelberg resistant to aminoglycosides and fosfomycin. Five isolates of *S.* Typhimurium and *S.* Heidelberg contained ARGs resistant to ≥5 antimicrobials (Hu et al., 2020). On the other hand, Rothrock et al. (2021) evaluated *Salmonella* spp. prevalence, diversity, and antimicrobial resistance profiles in antibiotic-free management systems of poultry flocks. A total of 2,305 samples were analyzed, with 337 *Salmonella* isolated and subjected to diversity analysis. The most prevalent serovars were *S.* Kentucky, *S.* Indiana, and *S.* Infantis. Even in an antibiotic-free system, *Salmonella* spp. isolates exhibited resistance to various antimicrobials, tetracycline and streptomycin being the most common and 27.3% of the *Salmonella* spp. isolates were MDR (Rothrock et al., 2021).

#### 3.1.2 Central America

In Guatemala, local samples from 300 chicken carcasses across seven departments were evaluated for the presence of *Salmonella* spp. Of the total samples, 103 were *Salmonella* spp., and serovars were identified for 43 samples. The most prevalent serovars were *S.* Paratyphi B, *S.* Heidelberg, and *S.* Derby. Resistance to enrofloxacin (52%), tetracycline (41%), trimethoprim-sulfamethoxazole (38%), and streptomycin (36%) were observed. Additionally, 31% of the isolates exhibited an MDR profile (Jarquin et al., 2015).

In another study, 65 chicken meat and 171 cecum samples were collected from the largest urban area in Costa Rica. *Salmonella* was identified in 58.5% (38/65) of the meat samples and in 38% (65/171) of the cecum samples. Regarding antimicrobial resistance in *Salmonella* isolates, both meat and cecum samples showed similar patterns: 92% and 94% were resistant to nalidixic acid, 84% and 91% to chloramphenicol, 82% and 91% to ampicillin, and 74% and 59% to cefotaxime, respectively. The MDR profile was observed in 90% of the *Salmonella* isolates from chicken meat and in 94% of those from cecum samples; the most frequent serovars were *S.* Infantis, *S.* Anatum, and *S.* Kentucky (Molina et al., 2024).

#### 3.1.3 South America

In the Southern region of Brazil, from 104 isolates of *Salmonella* spp. from poultry samples, 10% (11/104) were identified as *S.* Typhimurium. These isolates showed MDR, showing resistance to ceftiofur (27%), gentamicin (55%), and enrofloxacin (18%). These antibiotics are routinely used in poultry production (Biffi et al., 2014). In the same region, Pandini et al. (2015) evaluated the occurrence and antimicrobial resistance profile of *Salmonella* spp. isolated from poultry farms. A total of 342 samples were analyzed, of which 11% (39/342) tested positive for *Salmonella* spp. and 19 different serovars were identified. The most common serovars were *S.* Heidelberg, *S.* Mbandaka, and *S.* Newport. Resistance was observed to tetracycline (31%), nalidixic acid (28%), cephalothin (23%), and ampicillin (21%). Moreover, 23% (9/39) of the *Salmonella* isolates (*S.* Heidelberg and *S.* Infantis) showed an MDR profile. On the other hand, Ziech et al. (2016) detected MDR *Salmonella* spp. in 86% (84/98) of isolates from different broiler processing plants. Sereno et al. (2017), evaluated the antimicrobial resistance of 22 *Salmonella* spp. isolated from frozen poultry carcasses in the same region of Brazil. All strains were considered MDR. Among them, 100% showed resistance to quinolones (nalidixic acid), sulfonamides, and tetracyclines; 82% to cephalosporins (ceftiofur); 77% to penicillin; 36% to aminoglycosides; and 14% to monobactams and sulfonamides associated with pyrimidines. In the southeast region of Brazil, 29 *Salmonella* spp. isolates were collected from different points along the poultry slaughter line and their respective slaughterhouses. *S.* Kentucky and *S.* Enteritidis were the most common serovars. Regarding the antimicrobial resistance profile of *Salmonella* spp., 86% of the samples were resistant to aztreonam and ampicillin, 72% to tetracycline, and 55% to amoxicillin-clavulanic acid and sulfazotrim. Furthermore, 24% of the samples showed resistance to seven antibiotics, 21% to eight antibiotics, and 14% to five antibiotics (Cortez et al., 2006).

In the same region, a study on Brazilian egg production included 2,008 fecal samples collected from 151 commercial layer farms. Among the 22 identified *Salmonella* serovars, *S.* Mbandaka, *S.* Braenderup, *S.* Senftenberg, and *S.* Tennessee were the most common. The highest resistance rates were for streptomycin (94%) and sulfonamides (85%), and the MDR profile was found in 41% of the isolates (Benevides et al., 2020). In turn, in a study of 230 *Salmonella* spp. isolates from slaughterhouses in the central-west, south-east, and southern regions of Brazil, serovars and antimicrobial susceptibility were tested. The most common serovar was *S.* Heidelberg, followed by *S.* Minnesota, and *S.* Saintpaul. Of these isolates, 67% were MDR, with *S.* Heidelberg being the most common MDR serovar (55%), followed by *S.* Minnesota (22%) and *S.* Saintpaul (15%) (Rodrigues et al., 2020).

In Colombia, a study evaluated the prevalence of *Salmonella* spp. in 70 poultry farms, and 41% of these farms tested positive for *Salmonella*. The main serovars identified included *S.* Paratyphi B variant Java, and *S.* Heidelberg, with 100% and 92% of the isolates being MDR, respectively (Donado-Godoy et al., 2012). In another study in Colombia, 47 *Salmonella* spp. isolates from chicken carcasses were evaluated. All isolates were resistant to amikacin, gentamicin, tobramycin, cefoxitin, and cefazolin. Additionally, 42% of the isolates were found to be MDR, showing resistance to 11 or more antibiotics (Vélez et al., 2017). Another study from Colombia evaluated *Salmonella* spp. isolated from broiler farms, identifying two serovars from 49 chicken samples: *S.* Heidelberg and *S.* Paratyphi B. All *S.* Heidelberg isolates were resistant to ciprofloxacin and levofloxacin, and 7% were MDR, including enrofloxacin-resistant. Additionally, one of the isolates presented a gene conferring resistance to aminoglycosides. *S.* Paratyphi B showing resistance to ciprofloxacin was the most frequent, and only one isolate was MDR (Herrera-Sánchez et al., 2021).

In a study conducted in Peru, 13 isolates of *S.* Infantis were obtained from chicken meat. All isolates were MDR, showing resistance to ampicillin, nitrofurans, and quinolones (Martínez-Puchol et al., 2021). In Chile, 41 *S.* Infantis isolates obtained from various stages of a poultry meat production line were identified, characterized, and compared. All isolates were resistant to streptomycin, nalidixic acid, and tetracycline (Pardo-Esté et al., 2021). A study conducted in Trinidad and Tobago, involving 126 *Salmonella* spp. isolates recovered from 396 samples collected at four broiler processing plants, revealed *S.* Enteritidis, *S.* Javiana, and *S.* Infantis as the predominant serovars. Among the isolates, 91% exhibited antimicrobial resistance. Resistance was notably high to kanamycin (86%) and doxycycline (75%), while it was low to amoxicillin-clavulanic acid (2%) and trimethoprim-sulfamethoxazole (1%). Additionally, MDR was detected in 12% of the *Salmonella* spp. isolates (Khan et al., 2021).

### 3.2 Europe

Between 2001 and 2005, a significant number of MDR *Salmonella* spp. isolates were recovered from humans and poultry in Belgium and France. Among them, 11 *S.* Virchow isolates showed resistance to extended-spectrum cephalosporins. All these isolates showed additional resistance to trimethoprim-sulfamethoxazole and tetracycline, along with reduced susceptibility to ciprofloxacin (Bertrand et al., 2006; Cloeckaert et al., 2007). In another study, *S.* Virchow, along with *S.* Agona, *S.* Paratyphi B, and *S.* Typhimurium, exhibited resistance to extended-spectrum cephalosporins and chloramphenicol, florfenicol, streptomycin, spectinomycin, sulfonamide, tetracycline, and trimethoprim (Cloeckaert et al., 2007).

In Spain, a study evaluated the antimicrobial susceptibility of 59 *Salmonella* isolates from 226 chicken samples. *S.* Enteritidis, *S.* Infantis, *S.* Newport, and *S.* Typhimurium were the most prevalent. All isolates showed resistance to 3–13 antibiotics, with 22% being resistant to rifampicin, erythromycin, and nalidixic acid (Álvarez-Fernández et al., 2012).

A study from Italy reported 49 resistant isolates of *S.* Infantis with 70% MDR. The occurrence of resistance to high-priority antibiotics, such as quinolones, cephalosporins, and aminoglycosides, showed a mean resistance prevalence of 69%, 59%, and 26%, respectively (Franco et al., 2016). Recently, Castello et al. (2023) evaluated the prevalence and antimicrobial resistance of *Salmonella* spp. isolated from 145 poultry samples collected between 2019 and 2021. Forty *Salmonella* spp. were isolated and serotyping revealed *S.* Infantis, *S.* Kentucky, and *S.* Agona as the predominant serovars. Among the *S.* Infantis isolates, 80% were MDR. Regarding the MDR profile of all isolates, 9% were resistant to three different classes of antibiotics, 64% to four classes, and 27% to five classes. The most frequent MDR profiles showed resistance to cephalosporins, fluoroquinolones, sulfonamides, and tetracyclines (Castello et al., 2023).

In Switzerland, a study characterized 520 *S.* Infantis isolates from food (poultry meat), human infections, and environmental sources in 2010, 2013, and 2015. The authors performed antimicrobial susceptibility testing, and 75% of the isolates were MDR. Of these, 73% exhibited combined resistance to nalidixic acid, sulfamethoxazole, and tetracycline, with or without resistance to streptomycin (Hindermann et al., 2017). A study conducted in Portugal investigated seven *Salmonella* spp. isolates, including one *S.* Minnesota and six *S.* Heidelberg, which are uncommon in the region. All isolates were MDR, with 85% showing resistance to five different classes of antibiotics (quinolones, tetracyclines, sulfonamides, cephalosporins, and penicillins) (Campos et al., 2018).

An interesting study conducted in Germany characterized and compared *S.* Infantis isolates from different broiler farms in two distinct years (the 1990s and 2010). While all 12 isolates from the 1990s were susceptible to sulfamethoxazole, 83% (15/18) of the isolates from 2010 exhibited resistance to sulfamethoxazole, ciprofloxacin, tetracycline, nalidixic acid, and tigecycline (García-Soto et al., 2020). This resistance was attributed to the presence of the pESI-like megaplasmid, encoding antimicrobial resistance, and has been identified in other European countries, as well as in Israel (Aviv et al., 2014) and others (García-Soto et al., 2020).

Three broiler farms in England and Wales, which experienced an outbreak of MDR *S.* Infantis between 2013 and 2017, were studied longitudinally. Sixty *S.* Infantis isolates were tested for antimicrobial susceptibility, and all showed MDR to nalidixic acid, tetracycline, sulfonamide, streptomycin, and furazolidone. Furthermore, 78% (43/55) were resistant to trimethoprim-sulfamethoxazole. The authors emphasized that standard cleaning and disinfection protocols did not completely eradicate the infection (Newton et al., 2020).

In Poland, Ćwiek et al. (2020), evaluated *S.* Enteritidis isolates from poultry feces to assess resistance profiles, the prevalence of resistance genes, and their potential for biofilm formation. Fifty-one *S.* Enteritidis strains were isolated from the environments of chicken broiler, turkey broiler, and laying hen farms. Among these, 28% of the isolates from chicken samples exhibited the MDR phenotype, with the most common resistance pattern involving nalidixic acid, colistin, and sulfamethoxazole.

Finally, a significant amount of data on MDR *Salmonella* spp. has been collected and published in reports by the European Food Safety Authority (EFSA). For instance, the 2020 report (EFSA, 2020) highlighted that *Salmonella* spp. recovered from broiler carcasses exhibited high-to-extremely high resistance to ciprofloxacin and nalidixic acid, with overall resistance rates of 51% and 49%, respectively. MDR was observed at high levels in *Salmonella* spp. recovered from broiler carcasses (33%) and at moderate levels in *Salmonella* spp. recovered from turkey carcasses (15%). MDR among isolates from broiler carcasses varied, ranging from none in the United Kingdom to extremely high levels in Austria and Slovenia (87% and 91%, respectively).

Additionally, in their 2024 report (EFSA, 2024), high MDR was observed in *Salmonella* spp. recovered from broilers and turkeys in 2022 (44% and 40%, respectively). Among the *Salmonella* spp. recovered from poultry in 2022, resistance to colistin was commonly observed in *S.* Enteritidis isolates. This serovar accounted for 87% and 88% of colistin-resistant isolates recovered from broilers and layers, respectively. Overall resistance to ciprofloxacin was reported at a very high level in broilers (56%). *S.* Infantis accounted for most tigecycline-resistant isolates recovered from broilers (82%), layers (58%), and turkeys (48%).

### 3.3 Asia

A study conducted in Seoul, South Korea, on 100 chicken broiler carcass samples found that 42% were contaminated with *Salmonella* spp. Among them, *S.* Virchow, *S.* Bareilly, and *S.* Infantis were the most prevalent. Of the isolates, 76% were resistant to nalidixic acid, 71% were resistant to streptomycin, ampicillin, cefazolin, cephalothin, and cefotaxime, and 69% were resistant to tetracycline. Additionally, 29% of all samples contained MDR isolates resistant to five or more classes of antibiotics (Choi et al., 2015). A study in South Korea revealed the presence of 18 *Salmonella* spp. in 120 chicken samples, including *S.* Typhimurium, *S.* Hadar, and *S.* Rissen being the most frequent serovars. Furthermore, 28% of the isolates exhibited MDR profiles; the most common resistance patterns were streptomycin, fluoroquinolones, and tetracycline (Lee et al., 2016). In another study from South Korea, 82 *Salmonella* spp. were isolated from 853 poultry sources, revealing 33 MDR isolates. Ten more *Salmonella* spp., collected during a different period, showed MDR. MDR isolates showed the highest resistance to ampicillin (100%), nalidixic acid (77%), tetracycline (74%), and colistin (53%), and were attributed to five serovars: *S.* Enteritidis (58%), *S.* Agona (16%), *S.* Virchow (13%), *S.* Albany (9%), and *S.* Bareilly (2%). The highest resistance of *S.* Enteritidis was to ampicillin (100%), followed by nalidixic acid (96%), colistin (88%), and tetracycline (69%) (La et al., 2021).

A study in China across 24 provinces identified *S.* Typhimurium in chicken samples (food). The isolates showed MDR with high resistance to ampicillin, streptomycin, sulfonamides, and tetracycline (Yang et al., 2015). Other serovars have been isolated in China. Bai et al. (2016) observed a wide geographic distribution of *S.* Indiana, with 10% of chicken samples showing simultaneous resistance to ciprofloxacin and cefotaxime, indicating its establishment in some poultry flocks. The isolates showed ampicillin and ceftazidime resistance (Bai et al., 2016).

Zhang et al. (2016), studied antimicrobial resistance profiles of *Salmonella* isolates from chicken fecal swabs, identifying 69/159 MDR isolates, including *S.* Typhimurium, and *S.* Indiana. Resistance was most frequently observed to tetracycline (89%), ampicillin (84%), and gentamicin (81%). On the other hand, high resistance rates were observed for tetracycline (71%), sulfisoxazole (70%), ofloxacin (63%), chloramphenicol (48%), and florfenicol (47%), with 81% of isolates exhibiting MDR. *S.* Agona (18%), *S.* Corvallis (14%), *S.* Kentucky (12%), and *S.* Mbandaka (10%) were the dominant serovars in chicken samples (Zhang et al., 2018).

In Sichuan province, China, 189 of 627 broiler chicken samples tested positive for *Salmonella* spp., with *S.* Enteritidis and *S.* Typhimurium being predominant serovars. The isolates presented resistance to nalidixic acid (99%), ampicillin (88%), tetracycline (52%), ciprofloxacin (49%), trimethoprim-sulfamethoxazole (48%), and spectinomycin (34%), with 61% of isolates showing MDR (Zhu et al., 2017). In turn, Xiang et al. (2020) analyzed a salmonellosis outbreak linked to MDR *S.* Typhimurium found in food. Eight isolates were recovered from human samples, all exhibiting a similar MDR profile. Notably, the isolates demonstrated high levels of resistance to tetracycline, ampicillin, sulfisoxazole, nalidixic acid, and streptomycin. In another study, of the 1,237 chicken meat samples collected from five provinces in China (Beijing, Heilongjiang, Hubei, Jiangxi, and Shandong), 486 tested positives for *Salmonella* spp., with *S.* Enteritidis, *S.* Indiana, and *S.* Agona being the most prevalent. Furthermore, 36% of the isolates exhibited MDR; the dominant antimicrobial agents to which the isolates were resistant included ampicillin (64%), nalidixic acid (62%), and tetracycline (58%) (Wang et al., 2020).

Another study conducted in China assessed the resistance profiles of 105 *Salmonella* spp. recovered from deceased birds. Eight serovars were identified, with *S.* Enteritidis being the most prevalent. Antimicrobial resistance profiles revealed the following resistance rates: nalidixic acid (97.14%), ciprofloxacin (91%), ampicillin (71%), streptomycin (65%), and tetracycline (60%). Additionally, 77% of the *Salmonella* isolates were MDR (Li et al., 2022). In a study involving *S.* Indiana strains isolated from food, patients, healthy carriers, and the environment across 16 provinces of China, 45% of strains were derived from chicken and 10% from duck samples. The MDR profile was observed in 91% of the chicken and 87% of the duck samples (Zhang et al., 2022).

A study in Japan evaluated the prevalence and antimicrobial resistance in *Salmonella* spp. isolated from poultry meat sold in retail stores and poultry processing plants. Of the 512 samples, 56% were contaminated with *Salmonella* spp., resulting in 331 isolates, and 9 serovars were identified. The most frequent serovars were *S.* Infantis, *S.* Schwarzengrund, and *S.* Manhattan. Antimicrobial resistance was tested in 243 isolates (131 *S.* Infantis and 112 *S.* Schwarzengrund). The isolates showed high rates of resistance to tetracycline (81% in *S.* Infantis and 83.9% in *S.* Schwarzengrund), streptomycin (53% in *S.* Infantis and 77% in *S.* Schwarzengrund) and kanamycin (34% in *S.* Infantis and 82% in *S.* Schwarzengrund). Moreover, 66% of the isolates from *S.* Infantis and 86% from *S.* Schwarzengrund were resistant to two or more antimicrobial agents (Mori et al., 2018).

Duc et al. (2020), evaluated samples from broiler flocks in Kagoshima, Japan. From 192 flocks, 3071 samples of cecal specimens were obtained. The prevalence of *Salmonella* spp. was 8% (243/3071), and the serovars identified were *S.* Infantis (57%, 140/243), *S.* Manhattan (40%, 98/243), and *S.* Schwarzengrund (2%, 5/243). In terms of resistance rates, 95% showed resistance to streptomycin, 91% to oxytetracycline, 91% to sulfamethoxazole, 55% to ampicillin, 53% to cefotaxime, and 51% to ceftiofur. Moreover, 95% of the isolates exhibited MDR (Duc et al., 2020). On the other hand, Sasaki et al. (2021) evaluated the prevalence of *Salmonella* spp. in food workers and chicken samples. They characterized the isolates and tested their antimicrobial susceptibility. Among the chicken samples, *Salmonella* spp. was isolated in 85% (200/235). The most common serovars identified were *S.* Schwarzengrund, *S.* Infantis, and *S.* Manhattan. Furthermore, 87% of *Salmonella* isolates exhibited resistance to at least one antimicrobial, with 70% of strains resistant to two or more antimicrobials. The most frequent resistance was observed against streptomycin (73%), tetracycline (67%), and kanamycin (53%) (Sasaki et al., 2021).

A study from Russia reported three MDR *S.* Infantis showing resistance to more than eight antimicrobials. These isolates shared resistance to ciprofloxacin, enrofloxacin, doxycycline, tetracycline, spectinomycin, and trimethoprim. The MDR phenotype was linked to resistance genes carried by a pESI-like plasmid detected in the isolates (Bogomazova et al., 2020). In Qatar, the prevalence of *Salmonella* spp. in retail poultry was investigated, and the resistance profile was analyzed. From a total of 270 chicken carcasses collected from the three hypermarkets, 11% presented *Salmonella* spp. The isolates showed resistance to carbapenems (100%), cefepime (100%), piperacillin-tazobactam (100%), tetracycline (74%), nitrofurantoin (53%), ampicillin (50%), amoxicillin-clavulanic acid, ceftriaxone (27%), and ciprofloxacin (23%). In addition, 43% of the isolates showed MDR (Al-Hadidi et al., 2022).

In Iran, Manzari et al. (2022), identified 70 *Salmonella* spp. from 100 chicken samples, with the main serovars being *S.* Infantis, *S.* Enteritidis, and *S.* Paratyphi B. Among the *Salmonella* isolates, 74% showed MDR, mainly due to nalidixic acid, tetracycline, and trimethoprim-sulfamethoxazole without (13/70) and with azithromycin (12/70) (Manzari et al., 2022). In a study in Bangladesh, of the 55 samples (50 cloacal and 5 sewage), *Salmonella* spp. was identified in 76% of the cloacal swab samples and 100% of the sewage samples, and all isolates showed MDR. In the cloacal swab isolates, the greatest resistance was to polymyxin B and doxycycline (100%), nalidixic acid and colistin (97% each), perfloxacin and trimethoprim-sulfamethoxazole (84% each), ampicillin (74%), ciprofloxacin (68%), gatifloxacin (63%) and imipenem (45%) (Parvin et al., 2022).

Poultry production in India has been growing rapidly in the last decade, and scientific output on poultry-related *Salmonella* and its resistance is vast but still limited. A systematic review published by Shil et al. (2026) analyzed publications ranging from 2000 to 2025 in India and found high heterogeneity in *Salmonella* prevalence among regions, with dominant circulating serovars being *S.* Typhimurium, *S.* Enteritidis and *S.* Gallinarum, with emerging isolates showing prevalence as high as 43.1% for *S.* Infantis and 22.41% for *S.* Kentucky. Antimicrobial resistance data from across the country demonstrate a worsening trend over the decades, consistent patterns of which were found in another review from 2025 (Pooja Sajish et al., 2025). Tetracycline resistance in poultry samples went from 40% (2000–2010) to 55% (2011–2020) to 65% (2021–2025); beta-lactam resistance rose from 25% to 45% to 60%, in the same way; fluoroquinolone resistance from 15% to 35% to 55%; and cephalosporin from 10% to 30% to 50%. Resistance to colistin was confirmed in multiple regions, reaching 100% in isolates from North India and 59.1% in South India, where authors also found resistance to polymyxin B reaching 82% (Shil et al., 2026).

In addition, according to Sahu et al. (2025), NTS in India exhibits extensive MDR, showing an alarming temporal increase in resistance to critically important, frontline antimicrobials. Isolates demonstrate frequent resistance to nalidixic acid, ciprofloxacin, and third-generation cephalosporins (e.g., ceftriaxone, cefotaxime), alongside high resistance to ampicillin, amoxicillin, and erythromycin. Across non-human sources, *S.* Typhimurium and *S.* Enteritidis were identified as the most predominant serovars (Sahu et al., 2025).

#### 3.3.1 Southeast Asia

Among Southeast Asian (SEA) countries, the production and consumption of poultry is elevated due to cultural and religious influence. FAO data from 2023 estimate Indonesia’s chicken meat production to be over 4.04 million tons/year, followed by Thailand (1.83 million tons/year), Malaysia (1.6 million tons/year), Philippines (1.43 million tons/year) and finally Vietnam (1.07 million tons/year) (Imran-Arif et al., 2025).

Although this is a rather increasing economic market, SEA countries like Indonesia and Malaysia lack data regarding both the prevalence and resistance in poultry products, which makes our data toward some countries limited, but still worth discussing.

As previously stated, Indonesia is the largest producer of chicken meat among SEA countries, and even though it is considered to have a high consumption of antimicrobials in broiler production, proper investigation regarding resistant *Salmonella* contamination in the food chain has just begun (Coyne et al., 2020).

Takaichi et al. (2022) tested 50 *Salmonella* strains isolated from 120 samples of either chicken meat or duck rectal swabs across 12 of the most traditional markets in Surabaya, finding high prevalence of *S.* Schwarzengrund (23%), a considerably high resistance (at least 68% of all isolates resistant to one antibiotic) to nalidixic acid (60%), tetracycline (54%), and ampicillin (26%). *S.* Schwarzengrund was also found to be the serovar most likely to be MDR, with 12 strains (roughly 35.3%) resistant to antibiotics of 4 classes: penicillin, aminoglycoside, tetracycline and quinolones. Resistance genes were found in every non-susceptible strain, with *bla*_*TEM*_, *tetA/B* and mutations on *gyrA* accounting for the most frequent molecular findings. Plasmid-mediated quinolone resistance was not reported (Takaichi et al., 2022).

Later data from 2021 to 2022 found, in 150 chicken meat swab samples, only 17 (11.4%) positive isolates of *Salmonella* spp., mostly resistant to tetracycline (82%) and trimethoprim-sulfamethoxazole (59%), with five isolates MDR against three classes and one isolate resistant to four (Fanissa et al., 2022). Interestingly, data collected from the same year, but a different region presented a far larger prevalence of *Salmonella* (32/71 samples of chicken meat), but with similar resistance patterns: 84% resistant to oxytetracycline, 65% resistant to azithromycin and 53% resistant to sulfonamide trimethoprim, with 43% of all isolates presenting as MDR (Wibisono et al., 2025).

More recently, a study evaluating MDR in *Salmonella* and *E. coli* from duck meat found even more concerning data: all *Salmonella* spp. isolates (13/45 samples) were resistant to ceftaroline and nitrofurantoin, with high levels of resistance to doxycycline (92.3%), cefotaxime (84.6%) and both ceftriaxone and meropenem (53.8% each). Authors correlated this high level of resistance to ESBL genes such as *bla*_*TEM*_ and *bla*_*CTX–M*_ spreading worldwide, making MDR isolates account for 76.9% of all *Salmonella* isolates (Pisestyani et al., 2026).

On the other hand, data from 2024 from OECD and FAO classified Malaysia as the SEA country with the highest poultry meat consumption per capita in 2023 (29 kg/capita). With a large but often unregulated and informal market for poultry, concerns regarding the spread of AMR *Salmonella* at every step of the production chain led AMR surveillance to become one of the country’s top priorities (Imran-Arif et al., 2025). A 2016 study focusing on NTS prevalence found *Salmonella* in 39.7% (62/156) of all chicken meat samples collected from retails in Selangor and Negeri Sembilan, with high prevalence in wet markets (47%) and supermarkets (44%) (Shafini et al., 2017). In the same year, low prevalence and high resistance were found in another study conducted by Thung et al. (2016) from 120 samples of raw chicken meat collected from retails in Selangor, 36 were positive for *Salmonella*. Authors performed AST in all *S.* Enteritidis (*n* = 8) and *S.* Typhimurium (*n* = 3), resulting in all isolates being resistant to erythromycin, penicillin and vancomycin, minimal resistance to both nalidixic acid and streptomycin (9.09%) and 100% susceptibility to amoxicillin-clavulanic acid, gentamicin, tetracycline and trimethoprim (Thung et al., 2016).

High resistance to tetracycline and trimethoprim-sulfamethoxazole would be later reported by Abatcha et al. (2018). A slightly higher prevalence of *Salmonella* was found in chicken samples (17/35); most isolates were resistant to streptomycin (70.6%), sulfonamides (53%), and tetracycline (47%). Resistance to ampicillin, chloramphenicol, trimethoprim-sulphathiazole and nalidixic acid remained at 35.3%, with 12 (70.6%) isolates expressing an MDR profile. Authors also reported 6/17 isolates harboring class 1 integrons to be from chicken carcasses and performed PCR to check for AMR genes, but since data was not provided source-stratified, we restricted our analysis to phenotypical resistance only (Abatcha et al., 2018).

Another study found a high prevalence of *Salmonella* in samples from chicken meat, cloacal swabs and litter collected from supermarkets and poultry farms across Selangor (140/627). While the study focused specifically on colistin resistance, their results were notable 11.43% of *Salmonella* isolates showed phenotypic resistance to colistin (*n* = 16), and confirmation of *mcr* genes resulted in a single isolate harboring *mcr*-5, the first report of poultry-isolated *Salmonella* with this gene. Interestingly, authors found the sequence of the gene to be closely related to the *mcr*-5.3 gene variant recovered from *E. coli* isolates from horse samples in Brazil, pointing to a possible circulating of this gene (Karim et al., 2023).

Data regarding mobile genetic elements was addressed in a 2023 study from 63 environmental samples collected from poultry farms, *Salmonella* spp. was detected in 38.1% (*n* = 24). Higher levels of resistance (95.8%) were found for 2nd generation cephalosporins (cefazolin, cefuroxime and cefoxitin) and aminoglycosides (amikacin and gentamicin). Resistance to ampicillin was determined to be 62.5%, while ampicillin-sulbactam 50%, ciprofloxacin 33.8% and trimethoprim-sulfamethoxazole 12.5%. MDR was present in 16 out of the 24 isolates, and WGS performed in 8/24 resistant strains found class 1 integrons, transposons and several plasmids carrying aminoglycoside (*aph(3*′*)-Ia*), beta-lactam (*bla*_*TEM–*176_), sulfonamide (*dfrA14*, *sul3* and *tetA*), phenicol (*floR*) and quinolone (*qnrS1*) resistance genes (Syed Abu Thahir et al., 2023).

Later, Rajaratnam and Mahyudin (2024) published a study to assess both prevalence and AMR of *Salmonella* spp., and like what was previously found, out of 110 samples collected from retail markets in Seremban only 14 were positive for *Salmonella*, with every isolate being resistant to at least one of the 18 tested antibiotics. Higher resistance was found for erythromycin (92.9%), followed by ampicillin (78.6%) and chloramphenicol, aztreonam, tetracycline and trimethoprim-sulfamethoxazole (71.4%). While no resistance to ciprofloxacin, doxycycline and amoxicillin-clavulanic acid was found, 12/14 isolates were MDR and resistant to beta-lactams, with 42.9% resistant to five classes of antibiotics and 14.3% resistant to seven classes (Rajaratnam and Mahyudin, 2024).

A more recent study tested 790 samples from raw chicken meat for *Salmonella* across different areas of Peninsular Malaysia, detecting 135 isolates. Concurrently with previous studies, high resistance to erythromycin (87.41%) was reported along with tetracycline (85.19%) and sulfamethoxazole-trimethoprim (55.55%). Resistance to streptomycin and ampicillin were found in 29.63% of all isolates, and the lower rates of resistance were found for enrofloxacin (22.96%), nalidixic acid (17.04%), gentamicin (7.41%), cephalothin (5.9%), ceftriaxone (3.70%), amoxicillin-clavulanic acid (2.22%) and ciprofloxacin (0%). MDR was found in 93 isolates (68.88%), but overall, 132 isolates (97.78%) were resistant to at least one antibiotic (Ismail et al., 2024).

In Vietnam, a study of 148 chicken meat samples randomly collected from three northern provinces identified *Salmonella* spp. in 43% of the samples (63/148), comprising 13 serovars. The most prevalent serovars included *S.* Infantis and *S.* Anatum. All the isolates exhibited MDR and resistance to tetracycline (54%), sulfonamides (52%), streptomycin (41%), trimethoprim (36%), chloramphenicol (35%), and ampicillin (33%) was common (Hathai and Yamaguchi, 2012). The prevalence of antimicrobial-resistant *Salmonella* spp. isolates in raw meat and seafood samples from Vietnam were assessed in another study. Of the 409 raw meat samples, *Salmonella* was detected in 65% poultry samples, yielding 57 isolates from various serovars. The most prevalent serovars were *S.* Indiana, and *S.* Schwarzengrund. Resistance to one or more antimicrobials was observed in 90% of *Salmonella* from poultry, including resistance to tetracycline (74%), ampicillin (60%), chloramphenicol (46%), and nalidixic acid (42%) (Nguyen et al., 2016). Another study conducted with samples from three provinces in Vietnam assessed the prevalence of antimicrobial residues in chicken meat, the occurrence and serovar distribution of *Salmonella* spp., as well as antimicrobial resistance. Of the 119 chicken samples, 8% contained antimicrobial residues. Among 39 chicken meat samples, 72% were contaminated and 37 distinct *Salmonella* serovars were identified. The most prevalent were *S.* Kentucky, *S.* Anatum, and *S.* Rissen. MDR was the most common in *S.* Kentucky. The highest overall prevalence of resistance corresponded to quinolones, penicillins, and tetracyclines (Nhung et al., 2018).

### 3.4 Africa

Two studies conducted in geographically distinct regions of Egypt reported differences in the percentage of *Salmonella* spp. isolation, predominant serovars, and MDR profiles, despite being conducted within close periods (Elkenany et al., 2018, 2019). In the first study, Elkenany et al. (2018), detected *Salmonella* spp. in 13% (26/192) of broiler samples. The predominant serovars identified were *S.* Enteritidis, *S.* Kentucky, and *S.* Typhimurium. All isolates were resistant to trimethoprim-sulfamethoxazole (100%), amoxicillin-clavulanic acid and ampicillin (92%), tetracycline, streptomycin, and nalidixic acid (81%), and cefuroxime (58%). All MDR *Salmonella* spp. were resistant to ciprofloxacin and azithromycin. In another study, Elkenany et al. (2019) isolated 120 strains of *Salmonella* spp. from broiler chickens, the farm environment, and chicken carcasses. The serovars identified were *S.* Enteritidis, *S.* Typhimurium, and *S.* Kentucky. Antimicrobial susceptibility testing revealed absolute resistance to trimethoprim-sulfamethoxazole (100%), amoxicillin-clavulanic acid and ampicillin (68%), streptomycin (65%), doxycycline (40%), and cefaclor (37%). Moreover, 77% of *Salmonella* spp. showed MDR. In another study in Egypt, Elsayed et al. (2024), identified MDR in strains of *S.* Enteritidis isolated from broiler and layer chicken farms. *S.* Enteritidis strains were highly resistant to neomycin (100%), nalidixic acid, and cefoxitin (95%), norfloxacin (86%), cefotaxime (81%), amoxicillin (77%), amikacin (72%), and erythromycin (68%), chloramphenicol (40%), and tetracycline (31%) (Elsayed et al., 2024).

In Senegal, 53 *Salmonella* spp. were recovered from chicken carcasses sold at markets in Dakar, and 19 *Salmonella* spp. were isolated from human samples. The most common serovars were *S.* Brancaster, *S.* Kentucky, and *S.* Hadar. *Salmonella* spp. exhibited resistance to tetracycline (46%), trimethoprim-sulfamethoxazole (39%), erythromycin (33%), ciprofloxacin and nalidixic acid (17%), and ofloxacin (18%); 27.8% (20/72) of the isolates displayed MDR (Dieye et al., 2022). To investigate the possible sources of *Salmonella* spp. contamination in Burkina Faso, Kagambèga et al. (2018), examined the feces of different animals, including 350 poultry. *Salmonella* spp. was found in 55% of the chicken feces and 12% of the isolates showed resistance to one or more antimicrobials, and 1% of *S.* Typhimurium was classified as MDR (Kagambèga et al., 2018).

In southern Ethiopia, *Salmonella* prevalence was evaluated in three chicken centers. Forty-five *Salmonella* spp. strains were isolated from 270 poultry samples. All isolates were resistant to kanamycin and trimethoprim-sulfamethoxazole, nalidixic acid (97.8%), ampicillin (97%), cefoxitin (97%), streptomycin (97%), tetracycline (97%), chloramphenicol (91%), and ciprofloxacin (31%). Moreover, 93% (42/45) of the isolates were resistant to eight or more drugs, while all isolates were resistant to three or more drugs, showing MDR. *Salmonella* serovars and resistance genes were not reported in this work (Abdi et al., 2017).

In South Africa, environmental fecal samples were collected from chickens, ducks, and other animals in livestock production systems. Of the 361 samples, 54% were presumptive *Salmonella* spp. and 54% were confirmed as *Salmonella* spp. Most isolates were resistant to ampicillin (64%), tetracycline (63%), amoxicillin-clavulanate (49%), trimethoprim-sulfamethoxazole (38%) and ceftriaxone (20%). Additionally, 43% of the isolates were classified as MDR (Mthembu et al., 2019). Finally, a global synthesis matrix highlighting regional dominant serovars, common resistance phenotypes and possible drivers is summarized in Table 1.

**TABLE 1:** Global synthesis matrix, regional dominant serovars, common resistance phenotypes and possible drivers are summarized.

Region	MDR serovars	Phenotypic resistance	Genetic drivers	Primary selection pressures
North America	*S.* Kentucky	Tetracyclines	*bla*_*CTX–M–65*_	Commercial use of ceftiofur/tetracyclines
	*S.* Enteritidis	Ampicillin	pESI megaplasmid	Integrated industrial chains
	*S.* Infantis	Ceftiofur		
	*S.* Heidelberg	Cefotaxime		
South America	*S.* Heidelberg	Quinolones	Multidrug resistance plasmids	Widespread prophylactic use
	*S.* Minnesota	Tetracyclines	gyrA mutations	Fluoroquinolone/aminoglycoside dosing
	*S.* Infantis	Ceftiofur		
	*S.* Paratyphi B	Aminoglycosides		
Europe	*S.* Infantis	Ciprofloxacin	pESI-like megaplasmid	Post-ban therapeutic corrections
	*S.* Enteritidis	Nalidixic acid	Plasmid-mediated colistin	Biofilm persistence on commercial farms
	*S.* Typhimurium	Colistin		
	*S.* Virchow	Tigecycline		
Asia	*S.* Typhimurium	Ampicillin	*bla*_*TEM*_	Unregulated over-the-counter access
	*S.* Enteritidis	Ciprofloxacin	*bla*_*CTX–M*_	Fragmented federal biosecurity
	*S.* Indiana	Carbapenems	gyrA mutations	
		Colistin		
Southeast Asia	*S.* Schwarzengrund	Erythromycin	Class 1 integrons	Unregulated informal wet markets
	*S.* Infantis	Penicillins	*mcr* genes	Intense consumption per capita
	*S.* Kentucky	Doxycycline	*bla*_*TEM–176*_	
	*S.* Anatum	Meropenem	*qnrS1*	
Africa	*S.* Enteritidis	Trimethoprim-SMX	Chromosomal MDR	Lack of farm sanitation infrastructure
	*S.* Typhimurium	Amoxicillin-Clavulanate	Plasmid MDR profiles	Mixed-species containment
	*S.* Kentucky	Neomycin		
	*S.* Brancaster			

## 4 The pESI megaplasmid: genomic drivers of *Salmonella* Infantis proliferation

First report of a pESI-like plasmid in *Salmonella* Infantis is linked to Nógrády et al. (2007) study describing a multidrug-resistant *S.* Infantis present in 66% (91/138) of *S.* Infantis isolates from 2004 to 2005, from multiple sources including human, chicken feces, and chicken meat (Nógrády et al., 2007). Less than a decade later, Aviv et al. (2014) applied WGS and formally named the megaplasmid “pESI” (plasmid from emergent *Salmonella* Infantis), characterizing it by a combination of multiple antibiotic-resistance genes; heavy-metal resistance genes (via the *mer* operon); a siderophore iron-transport system (yersiniabactin: *ybt*, *fyu*A and *irp* genes) capable of biofilm formation; and a K-88 fimbria operon.

While MDR is a common trait of pESI-like plasmids, its genotype can vary and include the following: aminoglycoside resistance genes *aac(3)-Iva*, *ant(3*′′*)-Ia*, *aadA1*, *aph(4)-Ia*; beta-lactam resistance genes *bla*_*CTX–M–*65_, *bla*_*TEM*_; quinolone resistance via point mutations in *gyrA* and PMQR genes; trimethoprim resistance gene *dfrA14*; phenicol resistance gene *floR*; fosfomycin resistance gene *fosA3*; sulfonamide resistance gene *sul1*; and tetracycline resistance via *tetA*. Besides antimicrobial resistance, resistance to antiseptics through the quaternary ammonium compound gene *qacEdelta1* is commonly present (Aviv et al., 2014; dos Santos et al., 2022).

Comparative genomic analysis reveals no distinctive chromosomal characteristics in this *S.* Infantis strains; thus, its enhanced fitness and proliferation are presumably mediated by genes encoded on the pESI plasmid (Tyson et al., 2021). The pESI-like plasmid has also been reported in different serovars, as shown by an NCBI database screening performed by dos Santos et al. (2022). The authors screened 13,092 genomes from NCBI for sequences of the genes *ybtQ* and *ybtP*, previously described in *S.* Infantis carrying the pESI plasmid; after *in silico* genotyping they found 99.4% (*n* = 7,641) of the genomes belonged to *S.* Infantis, and 50 genomes belonged to other serovars: *S.* Agona (*n* = 26), *S.* Muenchen (*n* = 15), *S.* Schwarzengrund (*n* = 5), and *S.* Senftenberg (*n* = 4). Interestingly, all non-Infantis serovars were sampled not from poultry but from human sources and turkey meat (dos Santos et al., 2022). Thus, this indicates that the plasmid can spread to other *Salmonella* serovars both inside and outside the poultry industry.

Virulence genes on the pESI plasmid, particularly the yersiniabactin siderophore cluster (*fyuA*, *irp1*, *irp2*, *ybtA*, *ybtE*, *ybtP*, *ybtQ*, *ybtS*, *ybtT*, *ybtU*, *ybtX*), are part of the *Yersinia* high-pathogenicity island (HPI), a functional region widely disseminated among members of the family *Enterobacteriaceae* and contribute to the plasmid’s virulence in both Infantis and non-Infantis serovars, since they can be induced under iron-deficient conditions, greatly improving survival inside a host (Schubert et al., 2004). Chaperone-usher fimbriae are also encoded on the pESI plasmid, including a second, previously uncharacterized chaperone-usher fimbria cluster unique to this plasmid (designated *Ipf*, clustering *ipfA*, *ipfB*, *ipfC*, *ipfD*), in addition to a K88-like fimbria operon (including *feaD*, *feaE*, *feaG*, *feaC*, *feaF*, *feaH*, *feaI*, and regulatory genes *feaA*, *feaB*). Together, these fimbrial systems potentially enhance biofilm formation, adhesion to host cells, and even invasion into epithelial cells (Aviv et al., 2014). In addition, pESI-like plasmids have been reported widely in recent years. Poudel et al. (2026) conducted a population-dynamics analysis using the NCBI and PathogenFinder databases, obtaining 16,157 *S.* Infantis genomes; they found South America had the highest pESI prevalence (84.96%), followed by Asia (74.86%), North America (67.44%), Europe (67.44%), Africa (32.26%), and Australia (11.18%) (Poudel et al., 2026)

In conclusion, the global rise of *Salmonella* Infantis is driven not by chromosomal traits, but by the pESI megaplasmid (van der Voort et al., 2026). This mobile element equips strains with a robust toolkit of multidrug resistance, heavy-metal tolerances, and advanced virulence systems–like the yersiniabactin cluster and novel fimbriae–that maximize host colonization and biofilm formation. With global prevalence now exceeding 65% across the Americas, Asia, and Europe, pESI represents an escalating public health threat (Figure 2). Furthermore, its documented transmission into non-Infantis serovars from human and turkey sources underscores its dangerous adaptability, highlighting the critical need for expanded genomic surveillance across the food production industry.

## 5 Effects on resistance of *Salmonella* Infantis carrying pESI-like megaplasmid

The worldwide dissemination of MDR *Salmonella* Infantis has been strongly associated with the presence of the pESI-like megaplasmid (Kim and Lee, 2025), a large conjugative plasmid that enhances resistance to heavy metals and disinfectants, and also antimicrobial resistance through the carriage of the *bla*_*CTX–M*_ genes (dos Santos et al., 2022; Alba et al., 2023). Originally described in Israel and later disseminated worldwide, pESI-like plasmids are now frequently detected in *S.* Infantis isolates from South America, the United States, Europe, and Asia (Aviv et al., 2014; Kim and Lee, 2025; Vázquez et al., 2022). The *bla*_*CTX–M–*65_ positive lineage was initially identified in travelers returning from South American countries, suggesting this region as the probable origin of the lineage (Liao et al., 2023). Because *S.* Infantis can be isolated from retail poultry products imported from South America (Lee et al., 2021).

At the molecular level, pESI-like plasmids contain integrons associated with different AMR genes, which can accelerate the evolution from susceptible to resistant microorganisms (Alba et al., 2023), including *bla*_*CTX–M*_ genes associated with transposable elements that enable their acquisition and loss through transposition events, even among different strains or species (Russo et al., 2024). The pESI-like plasmid harbors a large diversity of transposable elements belonging to multiple families, including several copies of IS26 (Vázquez et al., 2022). Replicative transposition mediated by IS26 is prevalent in plasmids from clinical isolates and can result in extensive plasmid reorganization (He et al., 2015). Comparisons of the structure and gene content of pESI-like sequences indicate that these plasmids possess variable mosaic structures composed of regions inserted or transposed during multiple evolutionary events (Alba et al., 2023). The *bla*_*CTX–M–*65_ region is embedded within complex resistance cassettes containing genes associated with resistance to aminoglycosides, tetracyclines, sulfonamides, and phenicols, generating highly adaptable MDR genomic structures (Vázquez et al., 2022). In addition, pESI-like plasmids are self-transmissible and encode conjugation-associated genes (McMillan et al., 2020a,b; La et al., 2026), allowing horizontal transfer and potentially enhancing the dissemination of AMR genes among bacterial populations (McMillan et al., 2020b).

Recent genomic investigations have demonstrated that segments carrying *bla*_*CTX–M–*65_ can become integrated into the bacterial chromosome through IS26-mediated transposition events. For example, researchers in Taiwan identified the chromosomal insertion of a large resistance island containing *bla*_*CTX–M–*65_, *sul1*, and *tetA*, which likely originated from a plasmid-associated region (Liao et al., 2023). Another study reported chromosomal integration of *bla*_*CTX–M–*65_ in *S.* Infantis isolates recovered from retail poultry meat in the United States (Monte et al., 2025). In general, *Salmonella enterica* serovars appear to show a strong tendency toward chromosomal integration of antimicrobial resistance determinants (Guillemet and Lehtinen, 2026). This chromosomal integration may confer fitness advantages, such as reduced energetic costs and gradual adaptation of resistance genes to the host codon usage pattern, further stabilizing the multidrug-resistant phenotype (Fuzi, 2025). In conclusion, the remarkable success of the *bla*_*CTX–M–*65_/pESI-like lineage appears to result from the combination of efficient conjugative transfer, co-selection of multiple resistance traits, and enhanced ecological adaptation to poultry-associated environments, contributing to the rapid international expansion of this high-priority MDR clone (Figure 2).

In the case of resistance to heavy metals, according to Mourão et al. (2020), arsenic tolerance in multidrug-resistant *Salmonella* is highly prevalent in pig-associated serovars, specifically the epidemic European clone (ST34) of *S.* Typhimurium and its monophasic variant *S.* 4,[5],12:i:-, as well as emergent *S.* Rissen and *S.* Derby. Conversely, poultry-associated serovars like *S.* Enteritidis completely lack arsenic tolerance genes. Genotypically, the *arsBII* variant is strictly confined to the chromosome of the *S.* Typhimurium/ST34 clone, physically linked alongside copper and silver resistance clusters on an integrative conjugative element. Alternatively, the *acr3*-type operon is widely dispersed across 20 distinct serovars, predominantly embedded within the chromosome of *S.* Rissen and *S.* Derby (Mourão et al., 2020).

However, Wilson et al. (2019) evaluated 19 *Salmonella* strains isolated between 2001 and 2013 from Australian food production chains, that included samples of chicken carcass. The authors identified an ampicillin and streptomycin resistant *S.* Infantis carrying a plasmid harboring two arsenic resistance gene cassettes. The arsenic resistance cassette, *arsRCDAB*, that was also observed in two *S.* Singapore isolates with high tolerance to arsenate (Wilson et al., 2019). In this way, physical linkage on mobile genetic elements allows heavy metals to drive antibiotic resistance (Alvarez et al., 2023); thus, selective pressure from arsenic may have facilitated the co-selection and proliferation of pESI-positive *S.* Infantis clones (Schumann et al., 2025).

## 6 Role of international trade in spread of MDR *Salmonella*

International trade is one of the main pathways for the global dissemination of MDR bacterial pathogens, particularly *Salmonella enterica* associated with poultry products (Álvarez-Espejo et al., 2026). The expansion of global food supply chains, especially involving chicken meat, has increased both the movement of products and the risk of transmission of antimicrobial resistance (AMR) among pathogens (de Mesquita Souza Saraiva et al., 2022). As poultry meat is widely consumed worldwide and serves as an important reservoir for *Salmonella* (Rumi et al., 2025), this pathogen has been identified as exhibiting resistance to several antibiotics, with MDR strains being common (Sahu et al., 2025). The increasing dynamics of AMR in *Salmonella* align with the One Health framework (Mattock et al., 2024), in which a collaborative effort among multiple disciplines is necessary to maintain human and environmental health (McEwen and Collignon, 2018).

The poultry production sector is particularly relevant in this context because most of the antimicrobial use occurs in food production, where these compounds are widely applied for disease prevention and growth promotion (Zhao et al., 2024). Such practices exert strong selective pressure, which is inferred to lead to the emergence and diversification of resistant *Salmonella* strains (Mellor et al., 2019). These strains often exhibit resistance to multiple clinically important antibiotics, including ampicillin, chloramphenicol, and sulfamethoxazole–trimethoprim (Al-Hadidi et al., 2022), and *Salmonella* has also become resistant to tetracycline, with resistance to aminoglycosides increasing, as well as to third- and fourth-line antibiotics such as colistin and carbapenems (Kumar et al., 2025). The globalization of livestock trade influences antimicrobial use patterns across countries, shaping the dynamics of AMR and requiring coordinated international policies to prevent the amplification of resistance driven by interconnected production and trade systems (Avraam et al., 2021).

Evidence from importing countries further supports the role of trade in the spread of MDR *Salmonella*, as frequently reported in studies of imported poultry meat (Silveira et al., 2021; Li et al., 2025; Kamil et al., 2026). Antimicrobial-resistant *Salmonella* has been reported worldwide, with distribution across European countries, the United States of America, Canada, Australia, China, Chile, and several African countries (Wang et al., 2025). International trade and travel have become increasingly accessible to the general population due to globalization but have also contributed to the heightened risk of rapid dissemination of infectious diseases worldwide, including salmonellosis (Lamichhane et al., 2024).

Evidence from major poultry-exporting countries further supports the role of trade in the spread of MDR *Salmonella*, as reported in studies of chicken produced in Brazil and the United States, where antimicrobial-resistant *Salmonella* has been described in these production systems, often exhibiting resistance to multiple classes of clinically important antibiotics (Penha Filho et al., 2023; Ford et al., 2026). Brazil, India, Sri Lanka, and Turkey have reported widespread microbial contamination and MDR pathogens in poultry meat and eggs (Kamil et al., 2026). An outbreak in Europe, with over 200 cases, was traced to a pre-cooked frozen chicken meat product imported from a country outside the European Union (Gonzalez-Perez et al., 2025). These examples highlight how trade dynamics can act as sources of resistant strains that enter global circulation.

Overall, international trade plays a major role in both facilitating and intensifying the global dissemination of MDR *Salmonella*. Resistant bacteria can spread quickly across countries through international travel and trade, making antibiotic resistance a global issue (Cella et al., 2023). Among the sectors associated with this issue, livestock production, food animals, and their feed, particularly in animals that host resistant bacteria (Hanefeld et al., 2017). In this context, the One Health approach has been proposed as an important strategy to reduce the impact of AMR by integrating infection prevention and antimicrobial stewardship throughout the antimicrobial value chain, including research and development, production, regulatory authorization, distribution and supply, responsible use, and disposal practices (Altevogt et al., 2025). In conclusion, strengthening traceability systems and harmonizing surveillance programs among exporting and importing countries are essential to reduce the international spread of MDR pathogens through trade.

## 7 Final considerations on MDR *Salmonella*

The spotlight on MDR *Salmonella* spp. in poultry production worldwide, as highlighted in this review, shows that the most common serovars in the Americas are *S.* Heidelberg, *S.* Infantis, and *S.* Typhimurium. In Europe and Asia, the most common serovars are *S.* Infantis and *S.* Enteritidis, while in Africa, *S.* Enteritidis and *S.* Typhimurium are predominant. These observations are consistent with a previous study, confirming that different *Salmonella* serovars, including *S.* Enteritidis, *S.* Typhimurium, and *S.* Heidelberg, are frequently isolated in poultry production (Sodagari et al., 2023). These serovars are well-known causes of salmonellosis in humans and can be transmitted through the consumption of contaminated poultry products (Chanamé Pinedo et al., 2022), as *Salmonella* spp. has been detected in both laying hens (Pande et al., 2016; Wales and Davies, 2011) and broilers (Byrd et al., 1998; Shaji et al., 2023). Several studies have reported contamination from chicken viscera in slaughterhouses (Reiter et al., 2007), from chickens in Belgium (De Jong et al., 2014), and from broiler breeders and farms in Bangladesh (Barua et al., 2013), highlighting an increased risk of eventual transmission to humans.

The implementation of biosecurity measures on farms and in the industry has indeed contributed to the reduction in *Salmonella* spp. Biosecurity measures, such as controlling access to poultry farms, implementing strict hygiene practices, and monitoring and controlling potential sources of contamination, are essential to prevent the introduction and spread of *Salmonella* spp. in poultry production (Van Limbergen et al., 2018). In addition to these measures, the use of *Salmonella* vaccines in poultry flocks is important in reducing the spread of this microorganism (Desin et al., 2013).

In some cases, poultry flocks can be affected by bacterial infections caused by other species, such as avian pathogenic *Escherichia coli* (Ahmed et al., 2013). Historically, conventional treatment for bacterial infections in poultry has been a combination of sulfamethoxazole with trimethoprim (Baert et al., 2003) and a representative of the penicillin group (Huyghebaert et al., 2011). However, high resistance of *Salmonella* spp. to penicillin has made third- and fourth-generation cephalosporins more suitable for treating these infections (Yang et al., 2020). Nevertheless, the indiscriminate use of antimicrobials is concerning, and several studies have identified *Salmonella* spp. resistant to more than three classes of antimicrobials classified as MDR (Syed Abu Thahir et al., 2023; Aarestrup et al., 2007; Gieraltowski et al., 2016; Wright et al., 2005). As noted in the present review, in all continents, *Salmonella* MDR is present, and the resistance to various antibiotics that are used to treat infections in humans is a matter of great concern. Adopting a One Health perspective on *Salmonella enterica* as an emerging MDR pathogen in humans is becoming increasingly essential (Mattock et al., 2024).

This narrative review has some limitations related to the methodology used to evaluate *Salmonella* MDR across different studies. For example, some studies employed the minimum inhibitory concentration (MIC) assay, while others used the disk diffusion method for antimicrobial susceptibility testing. Furthermore, some authors assessed resistance genes exclusively through PCR or sequencing, while others combined susceptibility testing with molecular biology techniques. Moreover, several studies focused on specific classes of antimicrobials, potentially introducing bias in the findings. In other cases, some countries, such as China, a lot of these studies show similar findings and become redundant. On the other hand, the region, such as Africa has few studies available, which limits thorough analysis. Taking this information into account, methodically arranging the studies in the discussion is very difficult, for example, (i) Species (Poultry) - chicken and turkey studies, (ii) Stage of production - Preharvest studies, followed by post-harvest studies, followed by retail studies and then further processed poultry products, (iii) Product type - raw versus prepared products. However, these limitations do not undermine the overall conclusions, as this review offers an overview of MDR *Salmonella* spp. in poultry production over the past two decades.

## 8 Conclusion

1. Distribution of MDR across global poultry: over the past 26 years, NTS isolated from poultry sectors has demonstrated widespread and worsening MDR phenotypes. High-level and core resistance is universally observed against traditional veterinary classes, particularly tetracyclines, penicillins, and sulfonamides. Alarmingly, this resistance has extended rapidly to critically important human frontline therapies, including third- and fourth-generation cephalosporins, fluoroquinolones, and last-resort polymyxins (colistin), presenting a serious global threat to public health.
2. Geographical variations: globally, poultry-associated MDR *Salmonella* exhibits distinct regional distributions across continents. In North and Central America, *S.* Kentucky, *S.* Enteritidis, *S.* Heidelberg, and emerging *S.* Infantis clones dominate the production chain. Conversely, South American poultry networks are heavily characterized by high MDR rates in *S.* Heidelberg, *S.* Minnesota, and *S.* Infantis. Across Europe and Asia, *S.* Infantis and *S.* Enteritidis represent the primary public health threats, with *S.* Typhimurium and *S.* Indiana showing severe, concurrent resistance profiles in Asian flocks. Finally, *S.* Enteritidis and *S.* Typhimurium remain the predominant circulating MDR zoonotic serovars across African livestock systems.
3. High prevalence of *S.* Infantis: the international expansion and remarkable ecological fitness of *S.* Infantis within poultry systems are predominantly mediated by the acquisition of the pESI-like conjugative megaplasmid rather than distinct chromosomal modifications. This highly adaptable mobile genetic element serves as a consolidated molecular toolkit, linking complex antimicrobial resistance cassettes (such as *bla*_*CTX–M–*65_), heavy-metal tolerance genes (*mer* and *arsRCDAB* operon), advanced virulence clusters (yersiniabactin), and robust fimbrial biofilm systems, which allow the clone to survive aggressive cleaning and disinfection regimes on commercial farms.
4. International food trade and One Health implementations: Global food protein supply chains act as highly fluid pathways for the rapid international dissemination of zoonotic MDR *Salmonella* strains. Epidemiological patterns show a direct connection between the trade of imported poultry products and salmonellosis outbreaks in importing countries. Because agricultural antimicrobial selection pressures, environmental heavy-metal contamination, and human infectious risks are deeply interconnected, mitigating this global crisis requires harmonized international surveillance, strict cross-border traceability, and the integration of comprehensive One Health frameworks to manage antimicrobial stewardship uniformly across the value chain.

## References

[B1] Aarestrup F. M. Hendriksen R. S. Lockett J. Gay K. Teates K. McDermott P. F. et al. (2007). International spread of multidrug-resistant *Salmonella* Schwarzengrund in food products. *Emerg Infect Dis.* 13 726–731. 10.3201/eid1305.061489 17553251 PMC2738437

[B2] Abatcha M. G. Effarizah M. E. Rusul G. (2018). Prevalence, antimicrobial resistance, resistance genes and class 1 integrons of *Salmonella* serovars in leafy vegetables, chicken carcasses and related processing environments in Malaysian fresh food markets. *Food Control.* 91 170–180. 10.1016/j.foodcont.2018.02.039

[B3] Abdi R. D. Mengstie F. Beyi A. F. Beyene T. Waktole H. Mammo B. et al. (2017). Determination of the sources and antimicrobial resistance patterns of *Salmonella* isolated from the poultry industry in Southern Ethiopia. *BMC Infect. Dis.* 17:352. 10.1186/s12879-017-2437-2 28521744 PMC5437651

[B4] Ahmed A. M. Shimamoto T. Shimamoto T. (2013). Molecular characterization of multidrug-resistant avian pathogenic *Escherichia coli* isolated from septicemic broilers. *Int. J. Med. Microbiol.* 303 475–483. 10.1016/j.ijmm.2013.06.009 23891276

[B5] Alba P. Carfora V. Feltrin F. Diaconu E. L. Sorbara L. Dell’Aira E. et al. (2023). Evidence of structural rearrangements in ESBL-positive pESI(like) megaplasmids of S.Infantis. *FEMS Microbiol. Lett.* 370:fnad014. 10.1093/femsle/fnad014 36806934 PMC9990980

[B6] Al-Hadidi S. H. Al mana H. Almoghrabi S. Z. El-Obeid T. AlAli W. Q. Eltai N. O. (2022). Retail chicken carcasses as a reservoir of multidrug-resistant *Salmonella*. *Microbial Drug Resistance.* 28 824–831. 10.1089/mdr.2021.0414 35675669 PMC9347385

[B7] Altevogt B. M. Taylor P. Akwar H. T. Graham D. W. Ogilvie L. A. Duffy E. et al. (2025). A One Health framework for global and local stewardship across the antimicrobial lifecycle. *Commun. Med.* 5:414. 10.1038/s43856-025-01090-4 41057683 PMC12504729

[B8] Alvarez D. M. Barrón-Montenegro R. Conejeros J. Rivera D. Undurraga E. A. Moreno-Switt A. I. (2023). A review of the global emergence of multidrug-resistant *Salmonella enterica* subsp. enterica Serovar Infantis. *Int. J. Food Microbiol.* 403:110297. 10.1016/j.ijfoodmicro.2023.110297 37406596

[B9] Álvarez-Espejo D. M. Fredes-García D. Díaz-Gavidia C. Gutiérrez S. Barron-Montenegro R. Álvarez F. P. et al. (2026). Tracking antimicrobial resistance in *Salmonella* via poultry supply chains, human clinical samples, and environmental reservoirs. *Foods* 15:410. 10.3390/foods15030410 41682998 PMC12897130

[B10] Álvarez-Fernández E. Alonso-Calleja C. García-Fernández C. Capita R. (2012). Prevalence and antimicrobial resistance of *Salmonella* serotypes isolated from poultry in Spain: comparison between 1993 and 2006. *Int. J. Food Microbiol.* 153 281–287. 10.1016/j.ijfoodmicro.2011.11.011 22208955

[B11] Antunes P. Mourão J. Campos J. Peixe L. (2016). Salmonellosis: the role of poultry meat. *Clin. Microbiol. Infect.* 22 110–121. 10.1016/j.cmi.2015.12.004 26708671

[B12] Aviv G. Tsyba K. Steck N. Salmon-Divon M. Cornelius A. Rahav G. et al. (2014). A unique megaplasmid contributes to stress tolerance and pathogenicity of an emergent *Salmonella enterica* serovar Infantis strain. *Environ. Microbiol.* 16 977–994. 10.1111/1462-2920.12351 24320043

[B13] Avraam C. Lambrou A. S. Jiang W. Siddiqui S. (2021). Antimicrobial resistance and livestock trade for low and middle income countries: regional analysis of global coordination policies. *Front. Sustain. Food Syst.* 5:650315. 10.3389/fsufs.2021.650315

[B14] Baert K. De Baere S. Croubels S. De Backer P. (2003). Pharmacokinetics and oral bioavailability of sulfadiazine and trimethoprim in broiler chickens. *Vet. Res. Commun.* 27 301–309. 10.1023/A:1024084108803 12872830

[B15] Bai L. Zhao J. Gan X. Wang J. Zhang X. Cui S. et al. (2016). Emergence and diversity of *Salmonella enterica* serovar indiana isolates with concurrent resistance to ciprofloxacin and cefotaxime from patients and food-producing animals in China. *Antimicrob Agents Chemother.* 60 3365–3371. 10.1128/aac.02849-15 27001808 PMC4879380

[B16] Barrow P. Methner U. (2013). *Salmonella in Domestic Animals.* Wallingford: CABI.

[B17] Barua H. Biswas P. K. Olsen K. E. P. Shil S. K. Christensen J. P. (2013). Molecular characterization of motile serovars of *Salmonella enterica* from breeder and commercial broiler poultry farms in Bangladesh. *PLoS One* 8:e57811. 10.1371/journal.pone.0057811 23483931 PMC3590279

[B18] Benevides V. P. Rubio M. S. Alves L. B. R. Barbosa F. O. Souza A. I. S. Almeida A. M. et al. (2020). Antimicrobial resistance in *salmonella* serovars isolated from an egg-producing region in Brazil. *Braz. J. Poult. Sci.* 22 1–10. 10.1590/1806-9061-2020-1259

[B19] Bertrand S. Weill F. X. Cloeckaert A. Vrints M. Mairiaux E. Praud K. et al. (2006). Clonal emergence of extended-spectrum beta-lactamase (CTX-M-2)-producing *Salmonella enterica* serovar Virchow isolates with reduced susceptibilities to ciprofloxacin among poultry and humans in Belgium and France (2000 to 2003). *J. Clin. Microbiol.* 44 2897–2903. 10.1128/jcm.02549-05 16891509 PMC1594617

[B20] Bharat A. Mataseje L. Parmley E. J. Avery B. P. Cox G. Carson C. A. et al. (2022). One health genomic analysis of extended-spectrum β-lactamase-producing *Salmonella enterica*, Canada, 2012-2016. *Emerg. Infect. Dis.* 28 1410–1420. 10.3201/eid2807.211528 35731173 PMC9239887

[B21] Biffi C. P. Stefani L. M. Miletti L. C. Matiello C. A. Backes R. G. Almeida J. M. et al. (2014). Phenotypic and genotypic resistance profile of *Salmonella typhimurium* to antimicrobials commonly used in poultry. *Braz. J. Poult. Sci.* 16 93–96. 10.1590/1516-635x160293-96

[B22] Bogomazova A. N. Gordeeva V. D. Krylova E. V. Soltynskaya I. V. Davydova E. E. Ivanova O. E. et al. (2020). Mega-plasmid found worldwide confers multiple antimicrobial resistance in *Salmonella infantis* of broiler origin in Russia. *Int. J. Food Microbiol.* 319:108497. 10.1016/j.ijfoodmicro.2019.108497 31927155

[B23] Byrd J. A. Corrier D. E. Deloach J. R. Nisbet D. J. Stanker L. H. (1998). Horizontal transmission of *Salmonella typhimurium* in broiler chicks. *J. Appl. Poult. Res.* 7 75–80. 10.1093/japr/7.1.75

[B24] Caffrey N. Agunos A. Gow S. Liljebjelke K. Mainali C. Checkley S. L. (2021). *Salmonella* spp. prevalence and antimicrobial resistance in broiler chicken and turkey flocks in Canada from 2013 to 2018. *Zoonoses Public Health* 68 719–736. 10.1111/zph.12769 33780135

[B25] Campos J. Mourão J. Silveira L. Saraiva M. Correia C. B. Maçãs A. P. et al. (2018). Imported poultry meat as a source of extended-spectrum cephalosporin-resistant CMY-2-producing *Salmonella* Heidelberg and *Salmonella* Minnesota in the European Union, 2014–2015. *Int. J. Antimicrob. Agents* 51 151–154. 10.1016/j.ijantimicag.2017.09.006 28919197

[B26] Castello A. Piraino C. Butera G. Alio V. Cardamone C. Oliveri G. et al. (2023). Prevalence and antimicrobial resistance profiles of *Salmonella* spp. in poultry meat. *Ital. J. Food Saf.* 12:11135. 10.4081/ijfs.2023.11135 37405148 PMC10316271

[B27] CDC (2019). *Antibiotic Resistance Threats in the United States, 2019.* Ariyalur: Department of Health and Human Services, 138. 10.15620/cdc:82532

[B28] Cejas D. Vignoli R. Quinteros M. Marino R. Callejo R. Betancor L. et al. (2014). First detection of CMY-2 plasmid mediated β-lactamase in *Salmonella* Heidelberg in South America. *Rev. Argentina de Microbiol. / Argentinean J. Microbiol.* 46 30–33. 10.1016/S0325-7541(14)70044-6 24721271

[B29] Cella E. Giovanetti M. Benedetti F. Scarpa F. Johnston C. Borsetti A. et al. (2023). Joining forces against antibiotic resistance: the one health solution. *Pathogens* 12:1074. 10.3390/pathogens12091074 37764882 PMC10535744

[B30] Chanamé Pinedo L. Mughini-Gras L. Franz E. Hald T. Pires S. M. (2022). Sources and trends of human salmonellosis in Europe, 2015–2019: an analysis of outbreak data. *Int. J. Food Microbiol.* 379:109850. 10.1016/j.ijfoodmicro.2022.109850 35961158

[B31] Chattaway M. A. Langridge G. C. Wain J. (2021). *Salmonella* nomenclature in the genomic era: a time for change. *Sci. Rep.* 11:7494. 10.1038/s41598-021-86243-w 33820940 PMC8021552

[B32] Choi D. Chon J.-W. Kim H.-S. Kim D.-H. Lim J.-S. Yim J.-H. et al. (2015). Incidence, antimicrobial resistance, and molecular characteristics of nontyphoidal *Salmonella* including extended-spectrum β-lactamase producers in retail chicken meat. *J. Food Protect.* 78 1932–1937. 10.4315/0362-028X.JFP-15-145 26555514

[B33] Cloeckaert A. Praud K. Doublet B. Bertini A. Carattoli A. Butaye P. et al. (2007). Dissemination of an extended-spectrum-β-lactamase blaTEM-52 gene-carrying IncI1 plasmid in various *Salmonella enterica* serovars isolated from poultry and humans in Belgium and France between 2001 and 2005. *Antimicrob. Agents Chemother.* 51 1872–1875. 10.1128/aac.01514-06 17325216 PMC1855541

[B34] Cortez A. L. L. Carvalho A. C. Ikuno A. A. Bürger K. P. Vidal-Martins A. M. C. (2006). Resistência antimicrobiana de cepas de *Salmonella* spp. isoladas de abatedouros de aves. *Arquivos do Inst. Biol.* 73 157–163. 10.1590/1808-1657v73p1572006

[B35] Coyne L. Patrick I. Arief R. Benigno C. Kalpravidh W. McGrane J. et al. (2020). The costs, benefits and human behaviours for antimicrobial use in small commercial broiler chicken systems in Indonesia. *Antibiotics* 9:154. 10.3390/antibiotics9040154 32244693 PMC7235826

[B36] Crump J. A. Sjölund-Karlsson M. Gordon M. A. Parry C. M. (2015). Epidemiology, clinical presentation, laboratory diagnosis, antimicrobial resistance, and antimicrobial management of invasive *Salmonella* infections. *Clin. Microbiol. Rev.* 28 901–937. 10.1128/cmr.00002-15 26180063 PMC4503790

[B37] Ćwiek K. Korzekwa K. Tabiś A. Bania J. Bugla-Płoskońska G. Wieliczko A. (2020). Antimicrobial resistance and biofilm formation capacity of *Salmonella enterica* serovar enteritidis strains isolated from poultry and humans in Poland. *Pathogens* 9:643. 10.3390/pathogens9080643 32784631 PMC7459949

[B38] Davalos S. Santa-Cruz M. Condori R. Rodriguez J. Lucas J. R. (2025). Multiple antibiotic resistance of *Salmonella* Infantis in the Peruvian poultry production chain: detection in birds, the farming environment, and chicken carcasses. *Prevent. Vet. Med.* 234:106364. 10.1016/j.prevetmed.2024.106364 39510009

[B39] Davies N. Jørgensen F. Willis C. McLauchlin J. Chattaway M. A. (2022). Whole genome sequencing reveals antimicrobial resistance determinants (AMR genes) of *Salmonella enterica* recovered from raw chicken and ready-to-eat leaves imported into England between 2014 and 2019. *J. Appl. Microbiol.* 133 2569–2582. 10.1111/jam.15728 35880358 PMC9804530

[B40] De Jong A. Smet A. Ludwig C. Stephan B. De Graef E. Vanrobaeys M. et al. (2014). Antimicrobial susceptibility of *Salmonella* isolates from healthy pigs and chickens (2008–2011). *Vet. Microbiol.* 171 298–306. 10.1016/j.vetmic.2014.01.030 24598135

[B41] de Mesquita Souza Saraiva M. Lim K. do Monte D. F. M. Givisiez P. E. N. Alves L. B. R. de Freitas Neto O. C. et al. (2022). Antimicrobial resistance in the globalized food chain: a One Health perspective applied to the poultry industry. *Braz. J. Microbiol.* 53 465–486. 10.1007/s42770-021-00635-8 34775576 PMC8590523

[B42] Delgado-Suarez E. J. Puente-Cruz A. L. Sánchez-Zamorano L. M. Rubio-Lozano M. S. Ballesteros-Nova N. E. Hernández-Pérez C. F. et al. (2026). Emergence of multidrug-resistant blaCTX-M-65/gyrA_D87Y clones among the circulating *Salmonella* Infantis population in Mexico. *Microb. Genomics* 12 1–12. 10.1099/mgen.0.001645 41697755 PMC12908943

[B43] Desin T. S. Köster W. Potter A. A. (2013). *Salmonella* vaccines in poultry: past, present and future. *Expert Rev. Vacc.* 12 87–96. 10.1586/erv.12.138 23256741

[B44] Dieye Y. Hull D. M. Wane A. A. Harden L. Fall C. Sambe-Ba B. et al. (2022). Genomics of human and chicken *Salmonella* isolates in Senegal: broilers as a source of antimicrobial resistance and potentially invasive nontyphoidal salmonellosis infections. *PLoS One* 17:e0266025. 10.1371/journal.pone.0266025 35325007 PMC8947133

[B45] Donado-Godoy P. Gardner I. Byrne B. A. Leon M. Perez-Gutierrez E. Ovalle M. V. et al. (2012). Prevalence, risk factors, and antimicrobial resistance profiles of *Salmonella* from commercial broiler farms in two important poultry-producing regions of Colombia. *J. Food Protect.* 75 874–883. 10.4315/0362-028x.jfp-11-458 22564936

[B46] dos Santos A. M. P. Panzenhagen P. Ferrari R. G. Conte-Junior C. A. (2022). Large-scale genomic analysis reveals the pESI-like megaplasmid presence in *Salmonella* Agona, Muenchen, Schwarzengrund, and Senftenberg. *Food Microbiol.* 108:104112. 10.1016/j.fm.2022.104112 36088119

[B47] Doublet B. Praud K. Nguyen-Ho-Bao T. Argudín M. A. Bertrand S. Butaye P. et al. (2014). Extended-spectrum β-lactamase- and AmpC β-lactamase-producing d-tartrate-positive *Salmonella enterica* serovar Paratyphi B from broilers and human patients in Belgium, 2008–10. *J. Antimicrob. Chemother.* 69 1257–1264. 10.1093/jac/dkt504 24379303

[B48] Duc V. M. Shin J. Nagamatsu Y. Fuhiwara A. Toyofuku H. Obi T. et al. (2020). Increased *Salmonella* Schwarzengrund prevalence and antimicrobial susceptibility of *Salmonella enterica* isolated from broiler chickens in Kagoshima Prefecture in Japan between 2013 and 2016. *J. Vet. Med. Sci.* 82 585–589. 10.1292/jvms.20-0096 32213751 PMC7273603

[B49] Edirmanasinghe R. Finley R. Parmley E. J. Avery B. P. Carson C. Bekal S. et al. (2017). A whole-genome sequencing approach to study cefoxitin-resistant *Salmonella enterica* serovar Heidelberg isolates from various sources. *Antimicrob. Agents Chemother.* 61:e01919-16. 10.1128/aac.01919-16 28137797 PMC5365727

[B50] Elkenany R. Elsayed M. M. Zakaria A. I. El-sayed S. A.-E.-S. Rizk M. A. (2019). Antimicrobial resistance profiles and virulence genotyping of *Salmonella enterica* serovars recovered from broiler chickens and chicken carcasses in Egypt. *BMC Vet. Res.* 15:124. 10.1186/s12917-019-1867-z 31029108 PMC6486964

[B51] Elkenany R. M. Eladl A. H. El-Shafei R. A. (2018). Genetic characterisation of class 1 integrons among multidrug-resistant *Salmonella* serotypes in broiler chicken farms. *J. Glob. Antimicrob. Resist.* 14 202–208. 10.1016/j.jgar.2018.04.009 29684574

[B52] Elsayed M. M. El-Basrey Y. F. H. El-Baz A. H. Dowidar H. A. Shami A. Al-Saeed F. A. et al. (2024). Ecological prevalence, genetic diversity, and multidrug resistance of *Salmonella* Enteritidis recovered from broiler and layer chicken farms. *Poult. Sci.* 103:103320. 10.1016/j.psj.2023.103320 38215504 PMC10825688

[B53] Fanissa F. Effendi M. Tyasningsih W. Ugbo E. (2022). Multidrug-resistant *Salmonella* species from chicken meat sold at Surabaya Traditional Markets, Indonesia. *Biodiv. J. Biol. Div.* 23 2823–2829. 10.13057/BIODIV/D230606

[B54] Farhat M. Khayi S. Berrada J. Mouahid M. Ameur N. El-Adawy H. et al. (2024). *Salmonella enterica* serovar gallinarum biovars pullorum and gallinarum in poultry: review of pathogenesis, antibiotic resistance, diagnosis and control in the genomic era. *Antibiotics* 13:23. 10.3390/antibiotics13010023 38247582 PMC10812584

[B55] Folster J. P. Pecic G. Singh A. Duval B. Rickert R. Ayers S. et al. (2012). Characterization of extended-spectrum cephalosporin–resistant *Salmonella enterica* serovar heidelberg isolated from food animals, retail meat, and humans in the United States 2009. *Foodborne Pathog. Dis.* 9 638–645. 10.1089/fpd.2012.1130 22755514 PMC4620655

[B56] Ford L. Weller D. L. Steele M. K. Haro J. H. Ellison Z. Leeper M. et al. (2026). Trends in a persistent strain of multidrug-resistant *Salmonella* Infantis (REPJFX01) in humans and chickens — United States, 2010–2023. *J. Food Protect.* 89:100763. 10.1016/j.jfp.2026.100763 41887572 PMC13456058

[B57] Franco A. Leekitcharoenphon P. Feltrin F. Alba P. Cordaro G. Iurescia M. et al. (2016). Emergence of a clonal lineage of multidrug-resistant ESBL-producing *Salmonella* Infantis transmitted from broilers and broiler meat to humans in Italy between 2011 and 2014. *PLoS One* 10:e0144802. 10.1371/journal.pone.0144802 26716443 PMC4696813

[B58] Fuzi M. (2025). The fitness connection of antibiotic resistance. *Front. Microbiol.* 16:1556656. 10.3389/fmicb.2025.1556656 40276228 PMC12020126

[B59] García-Soto S. Abdel-Glil M. Y. Tomaso H. Linde J. Methner U. (2020). Emergence of multidrug-resistant *Salmonella enterica* subspecies enterica serovar infantis of multilocus sequence type 2283 in German broiler farms. *Front. Microbiol.* 11:1741. 10.3389/fmicb.2020.01741 32765483 PMC7380084

[B60] Gieraltowski L. Higa J. Peralta V. Green A. Schwensohn C. Rosen H. et al. (2016). National outbreak of multidrug resistant *Salmonella* Heidelberg infections linked to a single poultry company. *PLoS One* 11:e0162369. 10.1371/journal.pone.0162369 27631492 PMC5025200

[B61] Godínez-Oviedo A. Tamplin M. L. Bowman J. P. Hernández-Iturriaga M. (2020). *Salmonella enterica* in Mexico 2000–2017: epidemiology, antimicrobial resistance, and prevalence in food. *Foodborne Pathog. Dis.* 17 98–118. 10.1089/fpd.2019.2627 31647328

[B62] Gonzalez-Perez A. C. Landgren H. Vainio A. Kitowska W. Pihlajasaari A. Leinonen E. et al. (2025). A multi-country outbreak of *Salmonella* Mbandaka linked to pre-cooked, frozen chicken meat in ready-to-eat products, Finland, 2022 to 2023. *Euro Surveill.* 30:2400602. 10.2807/1560-7917.Es.2025.30.17.2400602 40314153 PMC12046970

[B63] Guibourdenche M. Roggentin P. Mikoleit M. Fields P. I. Bockemühl J. Grimont P. A. D. et al. (2010). Supplement 2003–2007 (No. 47) to the White-Kauffmann-Le Minor scheme. *Res. Microbiol.* 161 26–29. 10.1016/j.resmic.2009.10.002 19840847

[B64] Guillemet M. Lehtinen S. (2026). Bacterial strain structure shapes the trajectory of antibiotic resistance genes from plasmid to chromosome. *bioRxiv [Preprint].* 10.64898/2026.04.13.718102

[B65] Hanefeld J. Khan M. Tomson G. Smith R. (2017). Trade is central to achieving the sustainable development goals: a case study of antimicrobial resistance. *BMJ* 358:j3505. 10.1136/bmj.j3505 28739673 PMC5523143

[B66] Hathai T. Yamaguchi R. (2012). Molecular characterization of antibiotic-resistant *Salmonella* isolates from retail meat from markets in Northern Vietnam. *J. Food Protect.* 75 1709–1714. 10.4315/0362-028x.12-101 22947480

[B67] He S. Hickman A. B. Varani A. M. Siguier P. Chandler M. Dekker J. P. et al. (2015). Insertion sequence IS26 reorganizes plasmids in clinically isolated multidrug-resistant bacteria by replicative transposition. *mBio* 6:e00762. 10.1128/mbio.00762-15 26060276 PMC4471558

[B68] Hernandez S. M. Keel K. Sanchez S. Trees E. Gerner-Smidt P. Adams J. K. et al. (2012). Epidemiology of a *Salmonella enterica* subsp. enterica serovar Typhimurium strain associated with a songbird outbreak. *Appl. Environ. Microbiol.* 78 7290–7298. 10.1128/aem.01408-12 22885752 PMC3457103

[B69] Herrera-Sánchez M. P. Castro-Vargas R. E. Fandiño-de-Rubio L. C. Rodríguez-Hernández R. Rondón-Barragán I. S. (2021). Molecular identification of fluoroquinolone resistance in *Salmonella* spp. isolated from broiler farms and human samples obtained from two regions in Colombia. *Vet. World* 14 1767–1773. 10.14202/vetworld.2021.1767-1773 34475696 PMC8404129

[B70] Hindermann D. Gopinath G. Chase H. Negrete F. Althaus D. Zurfluh K. et al. (2017). *Salmonella enterica* serovar infantis from food and human infections, Switzerland, 2010–2015: poultry-related multidrug resistant clones and an emerging ESBL producing clonal lineage. *Front. Microbiol.* 8:1322. 10.3389/fmicb.2017.01322 28751886 PMC5507995

[B71] Hu L. Cao G. Brown E. W. Allard M. W. Ma L. M. Khan A. A. et al. (2020). Antimicrobial resistance and related gene analysis of *Salmonella* from egg and chicken sources by whole-genome sequencing. *Poult. Sci.* 99 7076–7083. 10.1016/j.psj.2020.10.011 33248624 PMC7705029

[B72] Huang J. Alzahrani K. O. Zhou G. Alsalman S. A. Alsufyani A. T. Alotaibi N. M. et al. (2025). Genomic survey of multidrug resistant *Salmonella enterica* serovar Minnesota clones in chicken products. *Npj Antimicrob. Resist.* 3:10. 10.1038/s44259-025-00077-4 39934234 PMC11814075

[B73] Huyghebaert G. Ducatelle R. Immerseel F. V. (2011). An update on alternatives to antimicrobial growth promoters for broilers. *Vet. J.* 187 182–188. 10.1016/j.tvjl.2010.03.003 20382054

[B74] Imran-Arif I. Kamaruzaman I. N. A. Mahamud S. N. A. Aklilu E. AbuBakar S. Loong S. K. (2025). Integrating One Health strategy to mitigate antibiotic resistance in *Salmonella*: insights from poultry isolates in Southeast Asia. *Trop. Biomed.* 42 27–35. 10.47665/tb.42.1.005 40163400

[B75] Ismail Z. Azmi N. N. Mahyudin N. A. Wan Omar W. H. Abdul Rahman M. Sapar M. (2024). *Salmonella* isolated from raw chicken meats at selected slaughterhouses in peninsular Malaysia; their antibiotic resistance profiles and biofilm formation on nutrient-limited media. *Malaysian Appl. Biol.* 53 55–71. 10.55230/mabjournal.v53i2.2767

[B76] Jajere S. M. (2019). A review of *Salmonella enterica* with particular focus on the pathogenicity and virulence factors, host specificity and antimicrobial resistance including multidrug resistance. *Vet. World* 12 504–521. 10.14202/vetworld.2019.504-521 31190705 PMC6515828

[B77] Jarquin C. Alvarez D. Morales O. Morales A. J. López B. Donado P. et al. (2015). *Salmonella* on raw poultry in retail markets in guatemala: levels, antibiotic susceptibility, and serovar distribution. *J. Food Protect.* 78 1642–1650. 10.4315/0362-028X.JFP-15-117 26319717

[B78] Jechalke S. Schierstaedt J. Becker M. Flemer B. Grosch R. Smalla K. et al. (2019). *Salmonella* establishment in agricultural soil and colonization of crop plants depend on soil type and plant species. *Front. Microbiol.* 10:967. 10.3389/fmicb.2019.00967 31156568 PMC6529577

[B79] Jeon H. Y. Kim Y. B. Lim S.-K. Lee Y. J. Seo K. W. (2019). Characteristics of cephalosporin-resistant *Salmonella* isolates from poultry in Korea, 2010–2017. *Poul. Sci.* 98 957–965. 10.3382/ps/pey418 30239919

[B80] Jung D. Morrison B. J. Rubin J. E. (2021). A review of antimicrobial resistance in imported foods. *Can. J. Microbiol.* 68 1–15. 10.1139/cjm-2021-0234 34570987

[B81] Kagambèga A. Lienemann T. Frye J. G. Barro N. Haukka K. (2018). Whole genome sequencing of multidrug-resistant *Salmonella enterica* serovar Typhimurium isolated from humans and poultry in Burkina Faso. *Trop. Med. Health* 46:4. 10.1186/s41182-018-0086-9 29449781 PMC5808401

[B82] Kamil Z. Lau W. S. Mohamed S. Stabler R. A. (2026). Multidrug-resistant non-typhoidal *Salmonella* and *Escherichia coli* in imported poultry products in the Maldives. *Access Microbiol.* 8:001114.v3. 10.1099/acmi.0.001114.v3 41836146 PMC12980945

[B83] Kanoatova S. Hurst M. Dougherty B. Dumoulin D. Silver H. M. O’Neill L. et al. (2024). Estimated reduction in human salmonellosis incidence in Canada from a new government requirement to reduce *Salmonella* in frozen breaded chicken products. *Epidemiol. Infect.* 152:e162. 10.1017/S0950268824001602 39648866 PMC11626448

[B84] Karim M. R. Zakaria Z. Hassan L. Faiz N. M. Ahmad N. I. (2023). The occurrence and molecular detection of mcr-1 and mcr-5 genes in *Enterobacteriaceae* isolated from poultry and poultry meats in Malaysia. *Front. Microbiol.* 14:1208314. 10.3389/fmicb.2023.1208314 37601372 PMC10435970

[B85] Khan A. S. Georges K. Rahaman S. Abebe W. Adesiyun A. A. (2021). Characterization of *Salmonella* isolates recovered from stages of the processing lines at four broiler processing plants in Trinidad and Tobago. *Microorganisms* 9:1048. 10.3390/microorganisms9051048 34068037 PMC8152471

[B86] Kim M. B. Lee Y. J. (2025). Genetic analysis of pESI-like megaplasmid in *Salmonella* Infantis from the poultry industry in Korea. *Vet. Microbiol.* 307:110576. 10.1016/j.vetmic.2025.110576 40494054

[B87] Kipper D. Mascitti A. K. De Carli S. Carneiro A. M. Streck A. F. Fonseca A. S. K. et al. (2022). Emergence, dissemination and antimicrobial resistance of the main poultry-associated *Salmonella* Serovars in Brazil. *Vet. Sci.* 9:405. 10.3390/vetsci9080405 36006320 PMC9415136

[B88] Klemm E. J. Shakoor S. Page A. J. Qamar F. N. Judge K. Saeed D. K. et al. (2018). Emergence of an extensively drug-resistant *Salmonella enterica* serovar typhi clone harboring a promiscuous plasmid encoding resistance to fluoroquinolones and third-generation cephalosporins. *mBio* 9:e00105-18. 10.1128/mbio.00105-18 29463654 PMC5821095

[B89] Kumar R. Adeyemi N. O. Chattaraj S. Alloun W. Thamarsha A. K. A. Anðelkoviæ S. et al. (2025). Antimicrobial resistance in *Salmonella*: one Health perspective on global food safety challenges. *Sci. One Health* 4:100117. 40687400 10.1016/j.soh.2025.100117 PMC12274912

[B90] La T.-M. Kim T. Lee H.-J. Lee J.-B. Park S.-Y. Choi I.-S. et al. (2021). Whole-genome analysis of multidrug-resistant *Salmonella* Enteritidis strains isolated from poultry sources in Korea. *Pathogens* 10:1615. 10.3390/pathogens10121615 34959570 PMC8707440

[B91] La T.-M. Kim T. Lee S.-W. Hyeon J.-Y. (2026). Complete genome sequences and structural variants of pESI-like plasmids in multidrug-resistant *Salmonella* Infantis carrying blaCTX-M-65 from retail chicken meat in South Korea. *J. Global Antimicrob. Resist.* 47 38–40. 41577246 10.1016/j.jgar.2026.01.005

[B92] Lamas A. Miranda J. M. Regal P. Vázquez B. Franco C. M. Cepeda A. (2018). A comprehensive review of non-enterica subspecies of *Salmonella enterica*. *Microbiol. Res.* 206 60–73. 10.1016/j.micres.2017.09.010 29146261

[B93] Lamichhane B. Mawad A. M. M. Saleh M. Kelley W. G. Harrington P. J. II Lovestad C. W. et al. (2024). Salmonellosis: an overview of epidemiology, pathogenesis, and innovative approaches to mitigate the antimicrobial resistant infections. *Antibiotics* 13:76. 10.3390/antibiotics13010076 38247636 PMC10812683

[B94] Larsson D. G. J. Flach C.-F. (2022). Antibiotic resistance in the environment. *Nat. Rev. Microbiol.* 20 257–269. 10.1038/s41579-021-00649-x 34737424 PMC8567979

[B95] Lee S.-H. Lee O.-M. Kang S.-I. Her M. Kang M.-S. Chae M. et al. (2025). Recent occurrence and rapid spread of multidrug-resistant *Salmonella* infantis in broiler flocks in Korea. *Foodborne Pathog. Dis.* 23 105–112. 10.1089/fpd.2024.0162 40014431

[B96] Lee S. K. Choi D. Kim H. S. Kim D. H. Seo K. H. (2016). Prevalence, seasonal occurrence, and antimicrobial resistance of *Salmonella* spp. isolates recovered from chicken carcasses sampled at major poultry processing plants of South Korea. *Foodborne Pathog. Dis.* 13 544–550. 10.1089/fpd.2016.2144 27442349

[B97] Lee W. W. Y. Mattock J. Greig D. R. Langridge G. C. Baker D. Bloomfield S. et al. (2021). Characterization of a pESI-like plasmid and analysis of multidrug-resistant *Salmonella enterica* Infantis isolates in England and Wales. *Microb. Genom.* 7:658. 10.1099/mgen.0.000658 34647862 PMC8627215

[B98] Li S. Zhan Z. Cui Y. Shi X. He S. (2025). Emergence of multidrug-resistant *Salmonella* Minnesota in chicken meat imported to China. *J. Fut. Foods*

[B99] Li Y. Kang X. -Dra A. Zhou X. Jia C. Müller A. et al. (2022). Genome-based assessment of antimicrobial resistance and virulence potential of isolates of non-pullorum/gallinarum *Salmonella* serovars recovered from dead poultry in China. *Microbiol. Spectr.* 10:e00965-22. 10.1128/spectrum.00965-22 35727054 PMC9431532

[B100] Liao Y. S. Wei H. L. Kuo H. C. Chen B. H. Wang Y. W. Teng R. H. et al. (2023). Chromosome-Borne CTX-M-65 extended-spectrum β-lactamase-producing *Salmonella enterica* serovar infantis, Taiwan. *Emerg. Infect. Dis.* 29 1634–1637. 10.3201/eid2908.230472 37486207 PMC10370839

[B101] Liu H. Whitehouse C. A. Li B. (2018). Presence and persistence of *Salmonella* in water: the impact on microbial quality of water and food safety. *Front. Public Health* 6:159. 10.3389/fpubh.2018.00159 29900166 PMC5989457

[B102] Magiorakos A. P. Srinivasan A. Carey R. B. Carmeli Y. Falagas M. E. Giske C. G. et al. (2012). Multidrug-resistant, extensively drug-resistant and pandrug-resistant bacteria: an international expert proposal for interim standard definitions for acquired resistance. *Clin. Microbiol. Infect.* 18 268–281. 10.1111/j.1469-0691.2011.03570.x 21793988

[B103] Manzari M. Fani F. Alebouyeh M. Moaddeli A. Rahnamaye Farzami M. Amin Shahidi M. et al. (2022). Multidrug-resistant *Salmonella* strains from food animals as a potential source for human infection in Iran. *Comp. Immunol. Microbiol. Infect. Dis.* 9:101898. 10.1016/j.cimid.2022.101898 36327760

[B104] Martínez-Puchol S. Riveros M. Ruidias K. Granda A. Ruiz-Roldán L. Zapata-Cachay C. et al. (2021). Dissemination of a multidrug resistant CTX-M-65 producer *Salmonella enterica* serovar Infantis clone between marketed chicken meat and children. *Int. J. Food Microbiol.* 344:109109. 10.1016/j.ijfoodmicro.2021.109109 33677191

[B105] Mattock J. Chattaway M. A. Hartman H. Dallman T. J. Smith A. M. Keddy K. et al. (2024). A one health perspective on *Salmonella enterica* serovar infantis, an emerging human multidrug-resistant pathogen. *Emerg. Infect. Dis.* 30 701–710. 10.3201/eid3004.231031 38526070 PMC10977846

[B106] McEwen S. A. Collignon P. J. (2018). Antimicrobial resistance: a one health perspective. *Microbiol. Spectr.* 6:10.1128/microbiolspec.arba-0009-2017. 10.1128/microbiolspec.arba-0009-2017 29600770 PMC11633550

[B107] McMillan E. A. Jackson C. R. Frye J. G. (2020a). Transferable plasmids of *Salmonella enterica* associated with antibiotic resistance genes. *Front. Microbiol.* 11:562181. 10.3389/fmicb.2020.562181 33133037 PMC7578388

[B108] McMillan E. A. Wasilenko J. L. Tagg K. A. Chen J. C. Simmons M. Gupta S. K. et al. (2020b). Carriage and gene content variability of the pESI-like plasmid associated with *Salmonella* infantis recently established in United States poultry production. *Genes* 11:1516. 10.3390/genes11121516 33352984 PMC7766811

[B109] Medrano H. Lee L. Young V. Janecko N. Deckert A. E. Gow S. P. et al. (2024). Surveillance of antimicrobial resistance in *Escherichia coli*, *Salmonella*, and Campylobacter recovered from laying hens, their environment and products in Canada indicated a stable level of resistance to critically important antimicrobials, in varying time periods between 2007 and 2021. *Int. J. Food Microbiol.* 412:110541. 10.1016/j.ijfoodmicro.2023.110541 38199015

[B110] Mellor K. C. Petrovska L. Thomson N. R. Harris K. Reid S. W. J. Mather A. E. (2019). Antimicrobial resistance diversity suggestive of distinct *Salmonella* Typhimurium sources or selective pressures in food-production animals. *Front. Microbiol.* 10:708. 10.3389/fmicb.2019.00708 31031720 PMC6473194

[B111] Mendonça E. P. Melo R. T. Oliveira M. R. M. Monteiro G. P. Peres P. A. B. M. Fonseca B. B. et al. (2020). Characteristics of virulence, resistance and genetic diversity of strains of *Salmonella* Infantis isolated from broiler chicken in Brazil. *Pesquisa Vet. Bras.* 40 29–38. 10.1590/1678-5150-PVB-5546

[B112] Mesa D. Almeida S. Krul D. Vasconcelos T. M. Siqueira A. C. da Silva Negoseki B. R. et al. (2026). An insight into the characterization of multidrug resistant *Salmonella* Minnesota isolated from poultry in Brazil. *Infect. Genet. Evol.* 139:105896. 10.1016/j.meegid.2026.105896 41679576

[B113] M’Ikanatha N. M. Sandt C. H. Localio A. R. Tewari D. Rankin S. C. Whichard J. M. et al. (2010). Multidrug-resistant *Salmonella* isolates from retail chicken meat compared with human clinical isolates. *Foodborne Pathog. Dis.* 7 929–934. 10.1089/fpd.2009.0499 20443729

[B114] Miranda J. M. Mondragón A. C. Martinez B. Guarddon M. Rodriguez J. A. (2009). Prevalence and antimicrobial resistance patterns of *Salmonella* from different raw foods in Mexico. *J. Food Protect.* 72 966–971. 10.4315/0362-028X-72.5.966 19517722

[B115] Molina A. Thye T. Muñoz-Vargas L. Zamora-Sanabria R. Chercos D. H. Hernández-Rojas R. et al. (2024). Molecular characterization of antibiotic resistant *Salmonella enterica* across the poultry production chain in Costa Rica: a cross-sectional study. *Int. J. Food Microbiol.* 416:110663. 10.1016/j.ijfoodmicro.2024.110663 38503221

[B116] Monte D. F. M. Harrell E. Harden L. Thakur S. (2025). Clonal spread of blaCTX-M-65 producing *Salmonella enterica* serovars detected in poultry retail meat in North Carolina, USA. *Sci. Rep.* 15:26520. 10.1038/s41598-025-02972-2 40691679 PMC12280192

[B117] Mori T. Okamura N. Kishino K. Wada S. Zou B. Nanba T. et al. (2018). Prevalence and antimicrobial resistance of *Salmonella* serotypes isolated from poultry meat in Japan. *Food Saf.* 6 126–129. 10.14252/foodsafetyfscj.2017019 32038899 PMC7004924

[B118] Mourão J. Rebelo A. Ribeiro S. Peixe L. Novais C. Antunes P. (2020). Tolerance to arsenic contaminant among multidrug-resistant and copper-tolerant *Salmonella* successful clones is associated with diverse ars operons and genetic contexts. *Environ. Microbiol.* 22 2829–2842. 10.1111/1462-2920.15016 32281716

[B119] Mthembu T. P. Zishiri O. T. El Zowalaty M. E. (2019). Molecular detection of multidrug-resistant *Salmonella* isolated from livestock production systems in South Africa. *Infect. Drug Resist.* 12 3537–3548. 10.2147/IDR.S211618 31814742 PMC6861519

[B120] Na S. H. Moon D. C. Kang H. Y. Song H.-J. Kim S.-J. Choi J.-H. et al. (2020). Molecular characteristics of extended-spectrum β-lactamase/AmpC-producing *Salmonella enterica* serovar Virchow isolated from food-producing animals during 2010–2017 in South Korea. *Int. J. Food Microbiol.* 322:108572. 10.1016/j.ijfoodmicro.2020.108572 32169770

[B121] Nadimpalli M. Fabre L. Yith V. Sem N. Gouali M. Delarocque-Astagneau E. et al. (2018). CTX-M-55-type ESBL-producing *Salmonella enterica* are emerging among retail meats in Phnom Penh, Cambodia. *J. Antimicrob. Chemother.* 74 342–348. 10.1093/jac/dky451 30376113

[B122] Nazir J. Manzoor T. Saleem A. Gani U. Bhat S. S. Khan S. et al. (2025). Combatting *Salmonella*: a focus on antimicrobial resistance and the need for effective vaccination. *BMC Infect. Dis.* 25:84. 10.1186/s12879-025-10478-5 39833704 PMC11744889

[B123] Newton K. Gosling B. Rabie A. Davies R. (2020). Field investigations of multidrug-resistant *Salmonella* Infantis epidemic strain incursions into broiler flocks in England and Wales. *Avian Pathol.* 49 631–641. 10.1080/03079457.2020.1809634 32783749

[B124] Nguyen D. T. Kanki M. Nguyen P. D. Le H. T. Ngo P. T. Tran D. N. et al. (2016). Prevalence, antibiotic resistance, and extended-spectrum and AmpC β-lactamase productivity of *Salmonella* isolates from raw meat and seafood samples in Ho Chi Minh City, Vietnam. *Int. J. Food Microbiol.* 236 115–122. 10.1016/j.ijfoodmicro.2016.07.017 27479779

[B125] Nhung N. T. Van N. T. B. Cuong N. V. Duong T. T. Q. Nhat T. T. Hang T. T. T. et al. (2018). Antimicrobial residues and resistance against critically important antimicrobials in non-typhoidal *Salmonella* from meat sold at wet markets and supermarkets in Vietnam. *Int. J. Food Microbiol.* 266 301–309. 10.1016/j.ijfoodmicro.2017.12.015 29275223 PMC5783717

[B126] Nógrády N. Tóth A. Kostyák A. Pászti J. Nagy B. (2007). Emergence of multidrug-resistant clones of *Salmonella* Infantis in broiler chickens and humans in Hungary. *J. Antimicrob. Chemother.* 60 645–648. 10.1093/jac/dkm249 17617553

[B127] Pande V. V. Devon R. L. Sharma P. McWhorter A. R. Chousalkar K. K. (2016). Study of *Salmonella* Typhimurium infection in laying hens. *Front. Microbiol.* 7:203. 10.3389/fmicb.2016.00203 26941727 PMC4766288

[B128] Pandini J. A. Pinto F. G. D. S. Muller J. M. Weber L. D. Moura A. C. D. (2015). Ocorrência e perfil de resistencia antimicrobiana de sorotipos de *Salmonella* spp. isolados de aviários do Paraná, Brasil. *Arquivos do Inst. Biol.* 82 1–6. 10.1590/1808-1657000352013

[B129] Pardo-Esté C. Lorca D. Castro-Severyn J. Krüger G. Alvarez-Thon L. Zepeda P. et al. (2021). Genetic characterization of *Salmonella* infantis with multiple drug resistance profiles isolated from a poultry-farm in chile. *Microorganisms* 9:2370. 10.3390/microorganisms9112370 34835497 PMC8621671

[B130] Parvin M. S. Ali M. Y. Mandal A. K. Talukder S. Islam M. T. (2022). Sink survey to investigate multidrug resistance pattern of common foodborne bacteria from wholesale chicken markets in Dhaka city of Bangladesh. *Sci. Rep.* 12:10818. 10.1038/s41598-022-14883-7 35752640 PMC9233690

[B131] Penha Filho R. A. C. Ferreira J. C. Galetti R. Kanashiro A. M. I. Berchieri A. Jr. da Costa Darini A. L. (2023). The rise of multidrug resistant *Salmonella* isolates in healthy chickens in Brazil by successful establishment of plasmid IncHI2A carrying several antibiotic resistance genes. *Braz. J. Microbiol.* 54 469–474. 10.1007/s42770-022-00893-0 36607526 PMC9944584

[B132] Perez-Sepulveda B. M. Hinton J. C. D. (2025). Microbe Profile: *Salmonella* Typhimurium: the master of the art of adaptation. *Microbiology* 171:1521. 10.1099/mic.0.001521 39786350 PMC11893365

[B133] Pisestyani H. Annisa R. F. Purnawarman T. (2026). Occurrence and multidrug resistance of *Salmonella* spp. and *Escherichia coli* from duck meat: implications for public health in Bogor, Indonesia. *IOP Confer. Series* 1603:012034. 10.1088/1755-1315/1603/1/012034

[B134] Pokharel B. M. Koirala J. Dahal R. K. Mishra S. K. Khadga P. K. Tuladhar N. R. (2006). Multidrug-resistant and extended-spectrum beta-lactamase (ESBL)-producing *Salmonella enterica* (serotypes Typhi and Paratyphi A) from blood isolates in Nepal: surveillance of resistance and a search for newer alternatives. *Int. J. Infect. Dis.* 10 434–438. 10.1016/j.ijid.2006.07.001 16978898

[B135] Pooja Sajish V. Uzzaman N. Aramvalarthan N. Asaduzzaman M. (2025). Prevalence, distribution and antimicrobial resistance profiles in poultry meat samples from India: a systematic review. *Front. Vet. Sci.* 12:1672628. 10.3389/fvets.2025.1672628 41246263 PMC12616860

[B136] Poudel S. Wang J. Bourassa D. (2026). Population dynamics and genomic characterization of *Salmonella* Infantis reveal poultry as a major reservoir of antimicrobial resistance genes and pESI megaplasmid. *Microbiol. Spectr.* 14:e04020-25. 10.1128/spectrum.04020-25 42023865 PMC13228007

[B137] Rajaratnam T. Mahyudin N. (2024). Antibiotic resistance of *Salmonella* spp. isolated from retail chicken meat in Seremban, N. Sembilan, Malaysia. *Malaysian J. Vet. Res.* 15 8–17.

[B138] Rajashekara G. Haverly E. Halvorson D. A. Ferris K. E. Lauer D. C. Nagaraja K. V. (2000). Multidrug-resistant *Salmonella* Typhimurium DT104 in Poultry. *J. Food Protect.* 63 155–161. 10.4315/0362-028X-63.2.155 10678417

[B139] Ramirez-Hernandez A. Carrascal-Camacho A. K. Varón-García A. Brashears M. M. Sanchez-Plata M. X. (2021). Genotypic characterization of antimicrobial resistant *Salmonella* spp. Strains from three poultry processing plants in Colombia. *Foods* 10:491. 10.3390/foods10030491 33668959 PMC7996530

[B140] Regalado-Pineda I. D. Rodarte-Medina R. Resendiz-Nava C. N. Saenz-Garcia C. E. Castañeda-Serrano P. Nava G. M. (2020). Three-Year longitudinal study: prevalence of *Salmonella Enterica* in chicken meat is higher in supermarkets than wet markets from Mexico. *Foods* 9:264. 10.3390/foods9030264 32121659 PMC7143798

[B141] Reiter M. G. R. Fiorese M. L. Moretto G. López M. C. Jordano R. (2007). Prevalence of *Salmonella* in a poultry slaughterhouse. *J. Food Protect.* 70 1723–1725. 10.4315/0362-028X-70.7.1723 17685349

[B142] Rodrigues I. Silva R. L. Menezes J. Machado S. C. A. Rodrigues D. P. Pomba C. et al. (2020). High prevalence of multidrug-resistant nontyphoidal *Salmonella* Recovered from broiler chickens and chicken carcasses in Brazil. *Braz. J. Poult. Sci.* 22 1–6. 10.1590/1806-9061-2019-1206

[B143] Rothrock M. J. Guard J. Y. Oladeinde A. (2021). *Salmonella* diversity along the farm-to-fork continuum of pastured poultry flocks in the Southeastern United States. *Front. Anim. Sci.* 2:761930. 10.3389/fanim.2021.761930 PMC11695693 39358927

[B144] Rumi M. A. Hasnine I. Sayeed M. A. Islam M. Dutta P. Uddin M. H. et al. (2025). Ecology and epidemiology of multidrug-resistant *Salmonella* in synanthropic small mammals in Bangladesh. *One Health* 21:101135. 10.1016/j.onehlt.2025.101135 40687595 PMC12275224

[B145] Russo I. Fischer J. Uelze L. Napoleoni M. Schiavano G. F. Andreoni F. et al. (2024). From farm to fork: spread of a multidrug resistant *Salmonella* Infantis clone encoding blaCTX-M-1 on pESI-like plasmids in Central Italy. *Int. J. Food Microbiol.* 410:110490. 10.1016/j.ijfoodmicro.2023.110490 37992554

[B146] Saggin B. F. Borges K. A. Furian T. Q. da Rosa Fünkler G. Mollerke R. Cenci M. M. et al. (2025). Highly resistant *Salmonella* Heidelberg circulating in broiler farms in southern Brazil. *Braz. J. Microbiol.* 56 723–729. 10.1007/s42770-024-01555-z 39500827 PMC11885699

[B147] Sahu A. A. Sephalika S. Mohakud N. K. Sahu B. R. (2025). Prevalence and multidrug resistance in non-typhoidal *Salmonella* in India: a 20-year outlook. *Acta Microbiol. Hellenica.* 70:6. 10.3390/amh70010006

[B148] Sanad Y. M. Johnson K. Park S. H. Han J. Deck J. Foley S. L. et al. (2016). Molecular Characterization of *Salmonella enterica* Serovars Isolated from a Turkey production facility in the absence of selective antimicrobial pressure. *Foodborne Pathog. Dis.* 13 80–87. 10.1089/fpd.2015.2002 26653998

[B149] Sasaki Y. Kakizawa H. Baba Y. Ito T. Haremaki Y. Yonemichi M. et al. (2021). Antimicrobial resistance in *Salmonella* isolated from food workers and chicken products in Japan. *Antibiotics* 10:1541. 10.3390/antibiotics10121541 34943753 PMC8698854

[B150] Schubert S. Rakin A. Heesemann J. (2004). The *Yersinia* high-pathogenicity island (HPI): evolutionary and functional aspects. *Int. J. Med. Microbiol.* 294 83–94. 10.1016/j.ijmm.2004.06.026 15493818

[B151] Schumann A. Wiedmann M. Orsi R. H. (2025). *Salmonella* serovar Infantis REPJFX01 isolates bear a pESI plasmid that includes additional genes not found in closely related non-REP strains. *Microb. Genomics* 11:001523. 10.1099/mgen.0.001523 41055947 PMC12504007

[B152] Sereno M. J. Ziech R. E. Druziani J. T. Pereira J. G. Bersot L. S. (2017). Antimicrobial susceptibility and biofilm production by *Salmonella* sp. strains isolated from frozen poultry carcasses. *Braz. J. Poult. Sci.* 19 103–108. 10.1590/1806-9061-2016-0268

[B153] Shafini A. B. Son R. Mahyudin N. A. Rukayadi Y. Tuan Zainazor T. C. (2017). Prevalence of *Salmonella* spp. in chicken and beef from retail outlets in Malaysia. *Int. Food Res. J.* 24 437–449.

[B154] Shaji S. Selvaraj R. K. Shanmugasundaram R. (2023). *Salmonella* infection in poultry: a review on the pathogen and control strategies. *Microorganisms* 11:2814. 10.3390/microorganisms11112814 38004824 PMC10672927

[B155] Shil S. Haldar S. Arora S. S. Pan D. Koley H. Chowdhury J. et al. (2026). Antimicrobial resistance in *Salmonella* from Indian poultry: trends, challenges, and one health perspectives. *J. Pure Appl. Microbiol.* 20 1–32. 10.22207/JPAM.20.1.31

[B156] Silveira L. Nunes A. Pista Â Isidro J. Belo Correia C. Saraiva M. et al. (2021). Characterization of multidrug-resistant isolates of *Salmonella enterica* Serovars Heidelberg and Minnesota from fresh poultry meat imported to portugal. *Microb. Drug Resist.* 27 87–98. 10.1089/mdr.2019.0384 32460607

[B157] Sodagari H. R. Shrestha R. D. Agunos A. Gow S. P. Varga C. (2023). Comparison of antimicrobial resistance among *Salmonella enterica* serovars isolated from Canadian turkey flocks, 2013 to 2021. *Poult. Sci.* 102:102655. 10.1016/j.psj.2023.102655 37030258 PMC10113892

[B158] Stanaway J. D. Parisi A. Sarkar K. Blacker B. F. Reiner R. C. Hay S. I. et al. (2019). The global burden of non-typhoidal *salmonella* invasive disease: a systematic analysis for the Global Burden of Disease Study 2017. *Lancet Infect. Dis.* 19 1312–1324. 10.1016/S1473-3099(19)30418-9 31562022 PMC6892270

[B159] Syed Abu Thahir S. Rajendiran S. Shaharudin R. Veloo Y. (2023). Multidrug-resistant *Salmonella* species and their mobile genetic elements from poultry farm environments in Malaysia. *Antibiotics* 12:1330. 10.3390/antibiotics12081330 37627750 PMC10451245

[B160] Takaichi M. Osawa K. Nomoto R. Nakanishi N. Kameoka M. Miura M. et al. (2022). Antibiotic resistance in non-typhoidal *Salmonella enterica* strains isolated from chicken meat in Indonesia. *Pathogens* 11:543. 10.3390/pathogens11050543 35631064 PMC9143091

[B161] Tate H. Folster J. P. Hsu C. H. Chen J. Hoffmann M. Li C. et al. (2017). Comparative analysis of extended-spectrum-β-lactamase CTX-M-65-producing *Salmonella enterica* serovar infantis isolates from humans, food animals, and retail chickens in the United States. *Antimicrob. Agents Chemother.* 61:e00488-17. 10.1128/aac.00488-17 28483962 PMC5487606

[B162] EFSA (2020). Union Summary Report on Antimicrobial Resistance in zoonotic and indicator bacteria from humans, animals and food in 2017/2018. *EFSA J.* 18:e06007. 10.2903/j.efsa.2020.6007 32874244 PMC7448042

[B163] EFSA (2024). Union summary report on antimicrobial resistance in zoonotic and indicator bacteria from humans, animals and food in 2021–2022. *EFSA J.* 22:e8583. 10.2903/j.efsa.2024.8583 38419967 PMC10900121

[B164] Thung T. Y. Mahyudin N. A. Basri D. F. Wan Mohamed Radzi C. W. J. Nakaguchi Y. Nishibuchi M. et al. (2016). Prevalence and antibiotic resistance of *Salmonella* Enteritidis and *Salmonella* Typhimurium in raw chicken meat at retail markets in Malaysia. *Poult. Sci.* 95 1888–1893. 10.3382/ps/pew144 27118863

[B165] Tiseo K. Huber L. Gilbert M. Robinson T. P. Van Boeckel T. P. (2020). Global trends in antimicrobial use in food animals from 2017 to 2030. *Antibiotics* 9:918. 10.3390/antibiotics9120918 33348801 PMC7766021

[B166] Tyson G. H. Li C. Harrison L. B. Martin G. Hsu C.-H. Tate H. et al. (2021). A multidrug-resistant *Salmonella* infantis clone is spreading and recombining in the United States. *Microb. Drug Resist.* 27 792–799. 10.1089/mdr.2020.0389 33232624 PMC11555764

[B167] Van Boeckel T. P. Brower C. Gilbert M. Grenfell B. T. Levin S. A. Robinson T. P. et al. (2015). Global trends in antimicrobial use in food animals. *Proc. Natl. Acad. Sci.* 112 5649–5654. 10.1073/pnas.1503141112 25792457 PMC4426470

[B168] van der Voort M. Castelijn G. A. A. Stassen J. H. M. (2026). Identification of *Salmonella* Infantis persistence in poultry products in the Netherlands with a role for the pESI plasmid. *Letters in Applied Microbiology* 79:ovag006. 10.1093/lambio/ovag006 41511445

[B169] Van Limbergen T. Dewulf J. Klinkenberg M. Ducatelle R. Gelaude P. Méndez J. et al. (2018). Scoring biosecurity in European conventional broiler production. *Poult. Sci.* 97 74–83. 10.3382/ps/pex296 29077940

[B170] Vázquez X. Fernández J. Rodríguez-Lozano J. Calvo J. Rodicio R. Rodicio M. R. (2022). Genomic Analysis of Two MDR Isolates of *Salmonella enterica* Serovar Infantis from a Spanish Hospital Bearing the blaCTX-M-65 Gene with or without fosA3 in pESI-like Plasmids. *Antibiotics* 11:786. 10.3390/antibiotics11060786 35740192 PMC9219668

[B171] Velasquez C. G. Macklin K. S. Kumar S. Bailey M. Ebner P. E. Oliver H. F. et al. (2018). Prevalence and antimicrobial resistance patterns of *Salmonella* isolated from poultry farms in southeastern United States. *Poult. Sci.* 97 2144–2152. 10.3382/ps/pex449 29608757

[B172] Velasquez-Munoz A. Castro-Vargas R. Cullens-Nobis F. M. Mani R. Abuelo A. (2024). Review: *Salmonella* Dublin in dairy cattle. *Front. Vet. Sci.* 10:1331767. 10.3389/fvets.2023.1331767 38264470 PMC10803612

[B173] Vélez D. C. Rodríguez V. García N. V. (2017). Phenotypic and genotypic antibiotic resistance of *Salmonella* from chicken carcasses marketed at Ibague, Colombia. *Braz. J. Poult. Sci.* 19 347–354. 10.1590/1806-9061-2016-0405

[B174] Wales A. D. Davies R. H. (2011). A critical review of *Salmonella* Typhimurium infection in laying hens. *Avian Pathol.* 40 429–436. 10.1080/03079457.2011.606799 21879803

[B175] Wang W. Zhao L. Hu Y. Dottorini T. Fanning S. Xu J. et al. (2020). Epidemiological study on prevalence, serovar diversity, multidrug resistance, and CTX-M-Type Extended-Spectrum β-Lactamases of *Salmonella* spp. from patients with diarrhea, food of animal origin, and pets in several provinces of China. *Antimicrob. Agents Chemother.* 64:e00092-20. 10.1128/aac.00092-20 32312775 PMC7318004

[B176] Wang Y. Xu X. Jia S. Qu M. Pei Y. Qiu S. et al. (2025). A global atlas and drivers of antimicrobial resistance in *Salmonella* during 1900-2023. *Nat. Commun.* 16:4611. 10.1038/s41467-025-59758-3 40382325 PMC12085583

[B177] World Health Organization [WHO] (2024). *WHO Bacterial Priority Pathogens List, 2024: Bacterial Pathogens of Public Health Importance to Guide Research, Development and Strategies to Prevent and Control Antimicrobial Resistance.* Geneva: WHO.

[B178] Wibisono F. J. Effendi M. H. Tyasningsih W. Rahmaniar R. P. Khairullah A. R. Kendek I. A. et al. (2025). Antibiotic resistance profiles of *Escherichia coli* and *Salmonella* spp. isolated from chicken meat sold in traditional markets in Gresik District, East Java, Indonesia. *Open Vet. J.* 15 2160–2170. 10.5455/OVJ.2025.v15.i5.34 40557094 PMC12184480

[B179] Wigley P. (2024). *Salmonella* and the chicken: reflections on salmonellosis and its control in the United Kingdom. *Poult. Sci. Manage.* 1:1. 10.1186/s44364-024-00001-y

[B180] Wilson A. Fox E. M. Fegan N. Kurtböke D. Í (2019). Comparative genomics and phenotypic investigations into antibiotic, heavy metal, and disinfectant susceptibilities of *Salmonella enterica* strains isolated in Australia. *Front. Microbiol.* 10:1620. 10.3389/fmicb.2019.01620 31379776 PMC6646423

[B181] Wright J. G. Tengelsen L. A. Smith K. E. Bender J. B. Frank R. K. Grendon J. H. et al. (2005). Multidrug-resistant *Salmonella* Typhimurium in four animal facilities. *Emerg. Infect. Dis.* 11 1235–1241. 10.3201/eid1108.050111 16102313 PMC3320505

[B182] Xiang Y. Li F. Dong N. Tian S. Zhang H. Du X. et al. (2020). Investigation of a salmonellosis outbreak caused by multidrug resistant *Salmonella* Typhimurium in China. *Front. Microbiol.* 11:801. 10.3389/fmicb.2020.00801 32411120 PMC7200987

[B183] Yang C. Li H. Zhang T. Chu Y. Zuo J. Chen D. (2020). Study on antibiotic susceptibility of *Salmonella typhimurium* L forms to the third and forth generation cephalosporins. *Sci. Rep.* 10:3042. 10.1038/s41598-020-59456-8 32080217 PMC7033113

[B184] Yang X. Wu Q. Zhang J. Huang J. Guo W. Cai S. (2015). Prevalence and characterization of monophasic *Salmonella* Serovar 1,4,[5],12:i:- of food origin in China. *PLoS One* 10:e0137967. 10.1371/journal.pone.0137967 26360603 PMC4567320

[B185] Zhang C.-Z. Ding X.-M. Lin X.-L. Sun R.-Y. Lu Y.-W. Cai R.-M. et al. (2019). The emergence of chromosomally located blaCTX-M-55 in *Salmonella* from foodborne animals in China. *Front. Microbiol.* 10:1268. 10.3389/fmicb.2019.01268 31231347 PMC6560199

[B186] Zhang L. Fu Y. Xiong Z. Ma Y. Wei Y. Qu X. et al. (2018). Highly prevalent multidrug-resistant *Salmonella* From chicken and pork meat at retail markets in Guangdong, China. *Front. Microbiol.* 9:2104. 10.3389/fmicb.2018.02104 30258419 PMC6143800

[B187] Zhang W.-H. Lin X.-Y. Xu L. Gu X.-X. Yang L. Li W. et al. (2016). CTX-M-27 producing *Salmonella enterica* serotypes typhimurium and indiana are prevalent among food-producing animals in China. *Front. Microbiol.* 7:436. 10.3389/fmicb.2016.00436 27065989 PMC4814913

[B188] Zhang Z. Chang J. Xu X. Hu M. He S. Qin X. et al. (2022). Phylogenomic analysis of *Salmonella enterica* Serovar Indiana ST17, an emerging multidrug-resistant clone in China. *Microbiol. Spectr.* 10:e00115-22. 10.1128/spectrum.00115-22 35862948 PMC9430114

[B189] Zhao C. Wang Y. Mulchandani R. Van Boeckel T. P. (2024). Global surveillance of antimicrobial resistance in food animals using priority drugs maps. *Nat. Commun.* 15:763. 10.1038/s41467-024-45111-7 38278814 PMC10817973

[B190] Zhu Y. Lai H. Zou L. Yin S. Wang C. Han X. et al. (2017). Antimicrobial resistance and resistance genes in *Salmonella* strains isolated from broiler chickens along the slaughtering process in China. *Int. J. Food Microbiol.* 259 43–51. 10.1016/j.ijfoodmicro.2017.07.023 28800411

[B191] Ziech R. E. Lampugnani C. Perin A. P. Sereno M. J. Sfaciotte R. A. P. Viana C. et al. (2016). Multidrug resistance and ESBL-producing *Salmonella* spp. isolated from broiler processing plants. *Braz. J. Microbiol.* 47 191–195. 10.1016/j.bjm.2015.11.021 26887244 PMC4822755

