# Supplementary material for: A spotlight on multidrug-resistant Salmonella in global poultry production
Source: Front Microbiol. 2026 Jul 1;17:1839142. doi: 10.3389/fmicb.2026.1839142 (PMC13377529; doi:10.3389/fmicb.2026.1839142)
Supplement: Supplementary file 1 [file Table_1.docx]

**Table 1.** ARGs identified in *Salmonella* strains from the poultry industry across countries. * Genes with variants not defined.

| **Antibiotic class** | **ARGs** | **Sample sources** | **Country** | **Reference** |
| --- | --- | --- | --- | --- |
| **Aminoglycosides** | *aadA* | Chicken | Brazil | Biffi et al., 2014 |
|  | *aadA1* | Chicken | Vietnam | Hathai et al., 2012 |
|  |  | Poultry | Israel | Aviv et al., 2014 |
|  |  | Chicken | Italy | Franco et al., 2016 |
|  |  | Chicken and turkey | Canada | Edirmanasinghe et al., 2017 |
|  |  | Poultry | Switzerland | Hindermann et al., 2017 |
|  |  | Chicken | United States | Tate et al., 2017 |
|  |  | Chicken | Burkina Faso | Kagambèga et al., 2018 |
|  |  | Chicken | Russia | Bogomazova et al., 2020 |
|  |  | Chicken and eggs | United States | Hu et al., 2020 |
|  |  | Chicken | Peru | Martínez-Puchol et al., 2021 |
|  |  | Poultry | Chile | Pardo-Esté et al., 2021 |
|  |  | Chicken | Colombia | Ramirez-Hernandez et al., 2021 |
|  |  | Chicken | United States | Tyson et al., 2021 |
|  |  | Chicken | Canada | Bharat et al., 2022 |
|  | *aadA2* | Turkey | United States | Sanad et al., 2016 |
|  |  | Chicken and turkey | Canada | Edirmanasinghe et al., 2017 |
|  |  | Chicken | Colombia | Vélez et al., 2017 |
|  |  | Chicken and eggs | United States | Hu et al., 2020 |
|  |  | Chicken | Canada | Bharat et al., 2022 |
|  |  | Chicken and duck | China | Li et al., 2022 |
|  | *aadA5* | Duck | Vietnam | Nguyen et al., 2016 |
|  |  | Chicken and duck | China | Li et al., 2022 |
|  | *aadA7* | Chicken | Senegal | Dieye et al., 2022 |
|  |  | Chicken and duck | China | Li et al., 2022 |
|  | *aadA16* | Chicken and duck | China | Li et al., 2022 |
|  | *aadB* | Chicken | Brazil | Biffi et al., 2014 |
|  |  | Chicken | Colombia | Vélez et al., 2017 |
|  | *ant(2”)-Ia* | Chicken | China | Zhu et al., 2017 |
|  | *ant(3”)-Ia* | Chicken | Germany | García-Soto et al., 2020 |
|  |  | Chicken | Japan | Sasaki et al., 2021 |
|  |  | Chicken and duck | China | Li et al., 2022 |
|  | *ant(3”)-Ia* | Chicken | Canada | Bharat et al., 2022 |
|  | *ant(3”)-Ib* |  |  |  |
|  | *aphA1* | Chicken | Japan | Duc et al., 2020 |
|  | *aphA1-1AB* | Chicken | Vietnam | Hathai et al., 2012 |
|  | *aph(3’)-Ia* | Chicken and eggs | United States | Hu et al., 2020 |
|  |  | Poultry | Korea | La et al., 2021 |
|  |  | Chicken | Colombia | Ramirez-Hernandez et al., 2021 |
|  |  | Poultry | Chile | Pardo-Esté et al., 2021 |
|  |  | Chicken and duck | China | Li et al., 2022 |
|  | *aph(3’)-Ib* | Poultry | Korea | La et al., 2021 |
|  | *aph(3’)-Ic* | Poultry | Switzerland | Hindermann et al., 2017 |
|  |  | Chicken | United States | Tate et al., 2017 |
|  | *aph(3’)-IIa* | Duck | Vietnam | Nguyen et al., 2016 |
|  |  | Chicken and duck | China | Li et al., 2022 |
|  | *aph(3”)-Ib* | Chicken and eggs | United States | Hu et al., 2020 |
|  |  | Chicken | China | Xiang et al., 2020 |
|  |  | Poultry | Korea | La et al., 2021 |
|  |  | Chicken | Senegal | Dieye et al., 2022 |
|  |  | Chicken and duck | China | Li et al., 2022 |
|  | *aph(4)-Ia* | Poultry | Switzerland | Hindermann et al., 2017 |
|  |  | Chicken | United States | Tate et al., 2017 |
|  |  | Poultry | Chile | Pardo-Esté et al., 2021 |
|  |  | Chicken | Colombia | Ramirez-Hernandez et al., 2021 |
|  |  | Chicken and duck | China | Li et al., 2022 |
|  | *aph(6)-Id* | Chicken and eggs | United States | Hu et al., 2020 |
|  |  | Poultry | Korea | La et al., 2021 |
|  |  | Chicken | Senegal | Dieye et al., 2022 |
|  | *aph(6)-Ic* | Chicken and eggs | United States | Hu et al., 2020 |
|  | *aph(6)-Id* | Chicken and duck | China | Li et al., 2022 |
|  | *aac(3)-VIa* | Chicken | Germany | García-Soto et al., 2020 |
|  | *aac(3’)-VIa* | Chicken | Canada | Bharat et al., 2022 |
|  | *aac(3’)-IIa* |  |  |  |
|  | *aac(6’)-Iy* |  |  |  |
|  | *aac(6’)-IIc* |  |  |  |
|  | *aac(3)-IVa* | Duck | Vietnam | Nguyen et al., 2016 |
|  |  | Poultry | Europe | Hindermann et al., 2017 |
|  |  | Chicken | United States | Tate et al., 2017 |
|  |  | Chicken and eggs | Switzerland | Hu et al., 2020 |
|  |  | Chicken | Colombia | Ramirez-Hernandez et al., 2021 |
|  |  | Chicken and duck | China | Li et al., 2022 |
|  |  | Chicken | Costa Rica | Molina et.al., 2024 |
|  | *aac(6’)-Iaa* | Chicken | Vietnam | Hathai et al., 2012 |
|  |  | Duck | Vietnam | Nguyen et al., 2016 |
|  |  | Chicken | Germany | García-Soto et al., 2020 |
|  |  | Chicken | China | Xiang et al., 2020 |
|  |  | Poultry | Korea | La et al., 2021 |
|  |  | Chicken | Japan | Sasaki et al., 2021 |
|  |  | Chicken and duck | China | Li et al., 2022 |
|  | *aac(6’)-Ib* | Chicken | Colombia | Herrera-Sánchez et al., 2021 |
|  | *aac(6’)-Ib-cr* | Chicken | China | Zhang et al., 2016 |
|  |  | Chicken and duck | China | Zhang et al., 2019 |
|  |  | Chicken | China | Xiang et al., 2020 |
|  |  | Chicken and duck | China | Li et al., 2022 |
|  | *aac(6’)-Id* | Chicken | China | Bai et al., 2016 |
|  |  | Chicken | China | Wang et al., 2020 |
|  |  | Chicken | Colombia | Ramirez-Hernandez et al., 2021 |
|  | *aaac(6)-Iy* | Poultry | Chile | Pardo-Esté et al., 2021 |
|  | *aac(6)-II* | Poultry | Israel | Aviv et al., 2014 |
|  | *strA* | Chicken | Canada | Bharat et al., 2022 |
|  | *strA* | Duck | Vietnam | Nguyen et al., 2016 |
|  | *strB* | Chicken and turkey | Canada | Edirmanasinghe et al., 2017 |
|  |  | Chicken | Burkina Faso | Kagambèga et al., 2018 |
|  | *strB* | Chicken | Colombia | Vélez et al., 2017 |
|  | *sat2* | Chicken | Colombia | Ramirez-Hernandez et al., 2021 |
|  | *armA* | Chicken and duck | China | Li et al., 2022 |
|  | *rmtB* | Chicken and duck | China | Li et al., 2022 |
|  |  | Chicken and duck | China | Zhang et al., 2022 |
|  | *aac(3)-IVa* | Chicken/Turkey | Mexico | Delgado-Suarez et al., 2022 |
|  | *aadA* | Chicken | Indonesia | Takaichi et al., (2022) |
|  | *aadA* | Poultry Environment | Malaysia | Abatcha et al., 2018 |
|  | *aac(6')-Iaa* | Poultry Environment | Malaysia | Syed Abu Thahir et al., 2023 |
| **Beta-lactams** | *blaTEM-1* | Poultry | Belgium | Bertrand et al., 2006 |
|  | *blaCTX-M-2* |  |  |  |
|  | *blaTEM-52* | Poultry | Belgium | Cloeckaert et al., 2007 |
|  | *blaCMY** | Chicken | United States | Folster et al., 2012 |
|  | *blaTEM** | Chicken | Vietnam | Hathai et al., 2012 |
|  | *blaOXA-1* |  |  |  |
|  | *blaPSE-1* |  |  |  |
|  | *blaOXA-21* | Poultry | Israel | Aviv et al., 2014 |
|  | *blaAmpC* | Chicken | Brazil | Biffi et al., 2014 |
|  | *blaCMY-2* |  |  |  |
|  | *blaCTX-M-1* | Chicken | Belgium | Doublet et al., 2014 |
|  | *blaCTX-M-2* |  |  |  |
|  | *blaCMY-2* |  |  |  |
|  | *blaTEM-1* |  |  |  |
|  | *blaCTX-M-1* | Chicken | Korea | Choi et al., 2015 |
|  | *blaCTX-M-15* |  |  |  |
|  | *blaCTX-M-14* | Chicken | China | Bai et al., 2016 |
|  | *blaCTX-M-65* |  |  |  |
|  | *blaCMY-2* |  |  |  |
|  | *blaCTX-M-1* | Chicken | Italy | Franco et al., 2016 |
|  | *blaCTX-M-15* | Chicken | Korea | Lee et al., 2016 |
|  | *blaTEM-1B* | Duck | Vietnam | Nguyen et al., 2016 |
|  | *blaCTX-M-14* | Chicken | China | Zhang et al., 2016 |
|  | *blaCTX-M-27* |  |  |  |
|  | *blaCTX-M-65* |  |  |  |
|  | *blaOXA-1* |  |  |  |
|  | *blaSHV-1* |  |  |  |
|  | *blaTEM-1b* |  |  |  |
|  | *blaTEM-1B* | Chicken and turkey | Canada | Edirmanasinghe et al., 2017 |
|  | *blaCMY-2* |  |  |  |
|  | *blaTEM** | Chicken | China | Zhu et al., 2017 |
|  | *blaCTX-M** |  |  |  |
|  | *blaTEM** | Chicken | Colombia | Vélez et al., 2017 |
|  | *blaCMY-2* | Chicken | Portugal | Campos et al., 2018 |
|  | *blaTEM** | Chicken | Egypt | Elkenany et al., 2018 |
|  | *blaTEM-1B* | Chicken | Burkina Faso | Kagambèga et al., 2018 |
|  | *blaCTX-M-15* | Chicken | China | Zhang et al., 2018 |
|  | *blaCTX-M-64* |  |  |  |
|  | *blaCTX-M-123* |  |  |  |
|  | *blaOXA-1* |  |  |  |
|  | *blaTEM-1* |  |  |  |
|  | *blaTEM-206* |  |  |  |
|  | *blaTEM-214* |  |  |  |
|  | *blaTEM-1* | Chicken and duck | South Africa | Mthembu et al., 2019 |
|  | *blaCMY-2* |  |  |  |
|  | *blaCTX-M-14* | Chicken | Russia | Bogomazova et al., 2020 |
|  | *blaTEM-1* | Chicken and eggs | United States | Hu et al., 2020 |
|  | *blaOXA-1* |  |  |  |
|  | *blaCMY* |  |  |  |
|  | *blaCMY-2* |  |  |  |
|  | *blaCARB-2* |  |  |  |
|  | *blaCTX-M-15* | Chicken | Korea | Na et al., 2020 |
|  | *blaCMY-2* |  |  |  |
|  | *blaAmpC* | Chicken | Brazil | Mendonça et al., 2020 |
|  | *blaCTX-M** |  |  |  |
|  | *blaSHV** |  |  |  |
|  | *blaCTX-M-65* | Chicken | China | Wang et al., 2020 |
|  | *blaCTX-M-130* |  |  |  |
|  | *blaTEM-1* | Chicken | China | Xiang et al., 2020 |
|  | *blaOXA-1* |  |  |  |
|  | *blaTEM-1B* | Poultry | Korea | La et al., 2021 |
|  | *blaCTX-M-65* | Chicken | Peru | Martínez-Puchol et al., 2021 |
|  | *blaDHA** |  |  |  |
|  | *blaTEM-1* | Chicken | Colombia | Ramirez-Hernandez et al., 2021 |
|  | *blaOXA-2* |  |  |  |
|  | *blaCTX-M-65* |  |  |  |
|  | *blaCMY-2* |  |  |  |
|  | *blaSHV-5* |  |  |  |
|  | *blaCMY-2* | Chicken | Japan | Sasaki et al., 2021 |
|  | *blaTEM-52B* |  |  |  |
|  | *blaCTX-M-1* | Chicken | Canada | Bharat et al., 2022 |
|  | *blaCTX-M-55* |  |  |  |
|  | *blaSHV-2* |  |  |  |
|  | *blaSHV-12* |  |  |  |
|  | *blaCMY-20* |  |  |  |
|  | *blaCTX-M-55* | Chicken and duck | China | Li et al., 2022 |
|  | *blaCTX-M-65* |  |  |  |
|  | *blaOXA-1* |  |  |  |
|  | *blaOXA-10* |  |  |  |
|  | *blaTEM-1B* |  |  |  |
|  | *blaCTX-M-65* | Chicken and duck | China | Zhang et al., 2022 |
|  | *blaOXA-1* |  |  |  |
|  | *blaCTX-M* | Chicken | Egypt | Elsayed et al., 2024 |
|  | *blaDHA-1* |  |  |  |
|  | *blaCTX-M-65* | Poultry | Switzerland | Hindermann et al., 2017 |
|  |  | Chicken | United States | Tate et al., 2017 |
|  |  | Poultry | Chile | Pardo-Esté et al., 2021 |
|  |  | Chicken | Costa Rica | Molina et.al., 2024 |
|  | *blaCMY-2* | Chicken | United States | M’Ikanatha et al., 2010 |
|  |  | Chicken | United States | Rothrock et al., 2021 |
|  | *blaCTX-M-65* | Chicken/Turkey | Mexico | Delgado-Suarez et al., 2022 |
|  | *blaTEM* | Chicken | Indonesia | Takaichi et al., (2022) |
|  | *blaTEM-1* | Poultry Environment | Malaysia | Abatcha et al., 2018 |
|  | *blaTEM-176* | Poultry Environment | Malaysia | Syed Abu Thahir et al., 2023 |
| **Macrolides** | *ermA* | Chicken | China | Xiang et al., 2020 |
|  | *ermB* |  |  |  |
|  | *ermR* |  |  |  |
|  | *Inu(F)* | Chicken | Colombia | Ramirez-Hernandez et al., 2021 |
|  |  | Chicken and duck | China | Li et al., 2022 |
|  | *mph* | Chicken | Colombia | Ramirez-Hernandez et al., 2021 |
|  | *mhpA* | Chicken | Canada | Bharat et al., 2022 |
|  |  | Chicken and duck | China | Li et al., 2022 |
|  |  | Chicken and duck | China | Zhang et al., 2022 |
|  | *ermA* | Chicken | Egypt | Elsayed et al., 2024 |
|  | *ermB* |  |  |  |
|  | *ermC* |  |  |  |
|  | *ermTR* |  |  |  |
|  | *mefA* |  |  |  |
|  | *msrA* |  |  |  |
|  | *mphA* | Poultry Environment | Malaysia | Syed Abu Thahir et al., 2023 |
| **Tetracyclines** | *tetA* | Chicken | Vietnam | Hathai et al., 2012 |
|  |  | Chicken | Italy | Franco et al., 2016 |
|  |  | Poultry | Switzerland | Hindermann et al., 2017 |
|  |  | Chicken | United States | Tate et al., 2017 |
|  |  | Chicken | Egypt | Elkenany et al., 2018 |
|  |  | Chicken | Germany | García-Soto et al., 2020 |
|  |  | Poultry | Korea | La et al., 2021 |
|  |  | Chicken | Japan | Sasaki et al., 2021 |
|  |  | Chicken and duck | China | Li et al., 2022 |
|  |  | Chicken | Egypt | Elsayed et al., 2024 |
|  | *tetA* | Chicken and turkey | Canada | Edirmanasinghe et al., 2017 |
|  | *tetB* | Chicken | Colombia | Ramirez-Hernandez et al., 2021 |
|  |  | Chicken | Senegal | Dieye et al., 2022 |
|  |  | Chicken | Costa Rica | Molina et.al., 2024 |
|  | *tetA* | Chicken and duck | South Africa | Mthembu et al., 2019 |
|  | *tetC* |  |  |  |
|  | *tetA* | Chicken | Russia | Bogomazova et al., 2020 |
|  | *tetM* |  |  |  |
|  | *tetR* |  |  |  |
|  | *tetB* | Chicken | China | Xiang et al., 2020 |
|  | *tetR* |  |  |  |
|  | *tetA* | Chicken | Canada | Bharat et al., 2022 |
|  | *tetB* |  |  |  |
|  | *tetD* |  |  |  |
|  | *tetA* | Poultry | Chile | Pardo-Esté et al., 2021 |
|  | *tetR* |  |  |  |
|  | *tetA* | Chicken and eggs | United States | Hu et al., 2020 |
|  | *tetB* |  |  |  |
|  | *tetC* |  |  |  |
|  | *tetG* |  |  |  |
|  | *tetB* | Chicken | Vietnam | Hathai et al., 2012 |
|  |  | Chicken | Burkina Faso | Kagambèga et al., 2018 |
|  |  | Chicken | Egypt | Elsayed et al., 2024 |
|  | *tetG* | Chicken | Vietnam | Hathai et al., 2012 |
|  | *tetL* | Chicken | Egypt | Elsayed et al., 2024 |
|  | *tetM* | Chicken | Egypt | Elsayed et al., 2024 |
|  | *tetA* | Chicken/Turkey | Mexico | Delgado-Suarez et al., 2022 |
|  | *tetA* | Chicken | Indonesia | Takaichi et al., (2022) |
|  | *tetA* | Poultry Environment | Malaysia | Abatcha et al., 2018 |
|  | *tetA* | Poultry Environment | Malaysia | Syed Abu Thahir et al., 2023 |
| **Fluoroquinolones** | *qnrA** | Chicken | China | Zhang et al., 2016 |
|  | *qnrB** |  |  |  |
|  | *qnrB5* | Chicken | Brazil | Campos et al., 2018 |
|  | *qnrB2* | Chicken and eggs | United States | Hu et al., 2020 |
|  | *qnrB19* |  |  |  |
|  | *qnrB** | Chicken | Colombia | Herrera-Sánchez et al., 2021 |
|  | *qnrB19* | Chicken | Colombia | Ramirez-Hernandez et al., 2021 |
|  | *qnrB2* | Chicken | Canada | Bharat et al., 2022 |
|  | *qnrB7* | Chicken | Senagal | Dieye et al., 2022 |
|  | *qnrB19* |  |  |  |
|  | *qnrB6* | Chicken and duck | China | Li et al., 2022 |
|  | *qnrD** | Chicken | China | Zhang et al., 2016 |
|  |  | Chicken | Cambodia | Nadimpalli et al., 2018 |
|  | *qnrD** | Chicken | China | Wang et al., 2020 |
|  | *qnrS** | Chicken | Egypt | Elkenany et al., 2018 |
|  |  | Chicken and duck | China | Zhang et al., 2019 |
|  | *qnrS1* | Chicken | Canada | Bharat et al., 2022 |
|  |  | Chicken and duck | China | Li et al., 2022 |
|  | *gyrA_D87Y* | Chicken/Turkey | Mexico | Delgado-Suarez et al., 2022 |
|  | *gyrA* | Chicken | Indonesia | Takaichi et al., (2022) |
|  | *qnrS1* | Poultry Environment | Malaysia | Syed Abu Thahir et al., 2023 |
| **Sulfonamides** | *sul1* | Chicken | Vietnam | Hathai et al., 2012 |
|  |  | Poultry | Israel | Aviv et al., 2014 |
|  |  | Chicken | Italy | Franco et al., 2016 |
|  |  | Poultry | Switzerland | Hindermann et al., 2017 |
|  |  | Chicken | United States | Tate et al., 2017 |
|  |  | Chicken | Egypt | Elkenany et al., 2018 |
|  |  | Chicken | Russia | Bogomazova et al., 2020 |
|  |  | Chicken | Germany | García-Soto et al., 2020 |
|  |  | Poultry | Chile | Pardo-Esté et al., 2021 |
|  |  | Chicken | Peru | Martínez-Puchol et al., 2021 |
|  |  | Chicken | Japan | Sasaki et al., 2021 |
|  |  | Chicken | Costa Rica | Molina et.al., 2024 |
|  | *sul1* | Chicken | Burkina Faso | Kagambèga et al., 2018 |
|  | *sul2* | Chicken and eggs | United States | Hu et al., 2020 |
|  |  | Chicken | China | Xiang et al., 2020 |
|  |  | Chicken | Colombia | Ramirez-Hernandez et al., 2021 |
|  |  | Chicken | Senegal | Dieye et al., 2022 |
|  | *sul1* | Chicken | Colombia | Ramirez-Hernandez et al., 2021 |
|  | *sul2* | Chicken | Canada | Bharat et al., 2022 |
|  | *sul3* | Chicken and duck | China | Li et al., 2022 |
|  | *sul1* | Chicken and turkey | Canada | Edirmanasinghe et al., 2017 |
|  | *sul3* |  |  |  |
|  | *sul2* | Chicken | Colombia | Vélez et al., 2017 |
|  |  | Chicken and duck | South Africa | Mthembu et al., 2019 |
|  |  | Poultry | Korea | La et al., 2021 |
|  | *sul1* | Chicken/Turkey | Mexico | Delgado-Suarez et al., 2022 |
|  | *sul1* | Poultry Environment | Malaysia | Abatcha et al., 2018 |
|  |  |  |  |  |
|  | *sul3* | Poultry Environment | Malaysia | Syed Abu Thahir et al., 2023 |
| **Trimetropim** | *dfrA1* | Poultry | Israel | Aviv et al., 2014 |
|  |  | Chicken | Colombia | Vélez et al., 2017 |
|  |  | Chicken | Burkina Faso | Kagambèga et al., 2018 |
|  |  | Poultry | Korea | La et al., 2021 |
|  | *dfrA7* | Chicken and duck | South Africa | Mthembu et al., 2019 |
|  | *dfrA14* | Poultry | Switzerland | Hindermann et al., 2017 |
|  |  | Chicken | United States | Tate et al., 2017 |
|  |  | Chicken | Russia | Bogomazova et al., 2020 |
|  |  | Chicken | Germany | García-Soto et al., 2020 |
|  |  | Poultry | Chile | Pardo-Esté et al., 2021 |
|  |  | Chicken | Japan | Sasaki et al., 2021 |
|  | *dfrA1* | Chicken | Vietnam | Hathai et al., 2012 |
|  | *dfrA12* |  |  |  |
|  | *dfrA1* | Chicken | Italy | Franco et al., 2016 |
|  | *dfrA14* |  |  |  |
|  | *dfrA12* | Chicken and eggs | United States | Hu et al., 2020 |
|  | *dfrA15* |  |  |  |
|  | *dfrA25* |  |  |  |
|  | *dfrA1* | Chicken | Colombia | Ramirez-Hernandez et al., 2021 |
|  | *dfrA7* |  |  |  |
|  | *dfrA1* | Chicken | Canada | Bharat et al., 2022 |
|  | *dfrA14* |  |  |  |
|  | *drfA18* |  |  |  |
|  | *dfrA1* | Chicken | Senegal | Dieye et al., 2022 |
|  | *dfrA7* |  |  |  |
|  | *dfrA14* |  |  |  |
|  | *dfrA15* |  |  |  |
|  | *dfrA1* | Chicken and duck | China | Li et al., 2022 |
|  | *dfrA14* |  |  |  |
|  | *dfrA17* |  |  |  |
|  | *dfrA27* |  |  |  |
|  | *dfrA** | Chicken | Egypt | Elkenany et al., 2018 |
|  | *dfrD** | Chicken | Egypt | Elsayed et al., 2024 |
|  | *dfrA14* | Chicken/Turkey | Mexico | Delgado-Suarez et al., 2022 |
|  | *dfrA14* | Poultry Environment | Malaysia | Syed Abu Thahir et al., 2023 |
| **Fosfomycin** | *fosA3* | Chicken | United States | Tate et al., 2017 |
|  |  | Chicken and duck | China | Li et al., 2022 |
|  |  | Chicken and duck | China | Zhang et al., 2022 |
|  | *fosA3* | Chicken | Colombia | Ramirez-Hernandez et al., 2021 |
|  | *fosA7* |  |  |  |
|  | *fosA** | Poultry | Switzerland | Hindermann et al., 2017 |
|  |  | Chicken and eggs | United States | Hu et al., 2020 |
|  |  | Poultry | Chile | Pardo-Esté et al., 2021 |
|  | *fosA7* | Chicken | Senegal | Dieye et al., 2022 |
|  | *fosA* | Chicken/Turkey | Mexico | Delgado-Suarez et al., 2022 |
|  | *fosA* | Poultry Environment | Malaysia | Syed Abu Thahir et al., 2023 |
| **Phenicol** | *floR** | Poultry | Switzerland | Hindermann et al., 2017 |
|  |  | Chicken | United States | Tate et al., 2017 |
|  |  | Poultry | Chile | Pardo-Esté et al., 2021 |
|  |  | Chicken | Vietnam | Hathai et al., 2012 |
|  |  | Chicken and eggs | United States | Hu et al., 2020 |
|  |  | Chicken | Canada | Bharat et al., 2022 |
|  |  | Chicken | Costa Rica | Molina et.al., 2024 |
|  | *cmlA1* | Chicken and turkey | Canada | Edirmanasinghe et al., 2017 |
|  | *catA1* | Chicken | Burkina Faso | Kagambèga et al., 2018 |
|  | *florR** | Chicken | Colombia | Ramirez-Hernandez et al., 2021 |
|  | *cmlA1* |  |  |  |
|  | *catA2* | Chicken and duck | China | Li et al., 2022 |
|  | *catB3* |  |  |  |
|  | *cmlA1* |  |  |  |
|  | *floR* |  |  |  |
|  | *floR* | Chicken/Turkey | Mexico | Delgado-Suarez et al., 2022 |
|  | *floR* | Poultry Environment | Malaysia | Abatcha et al., 2018 |
|  | *floR* | Poultry Environment | Malaysia | Syed Abu Thahir et al., 2023 |
| **Colistin** | *mcr-1* | Chicken | Malaysia | Karim et al., 2023 |
|  | *mcr-5* | Chicken | Malaysia | Karim et al., 2023 |
